# Glacier retreat shapes organic nutrient availability across lake succession

**DOI:** 10.1126/sciadv.aef3201

**Published:** 2026-08-07

**Authors:** Grégoire Saboret, Elliot Lewis, Nicolaj Krog Larsen, Antigoni Samourdani, Carsten Johnny Schubert, Jakob Brodersen

**Affiliations:** ^1^ Department of Surface Waters, Eawag: Swiss Federal Institute of Aquatic Science and Technology, 6047 Kastanienbaum, Switzerland.; ^2^ Department of Fish Ecology and Evolution, Eawag: Swiss Federal Institute of Aquatic Science and Technology, 6047 Kastanienbaum, Switzerland.; ^3^ Department of Aquatic Ecology & Evolution, Institute of Ecology and Evolution, University of Bern, 3012 Bern, Switzerland.; ^4^ Centre for GeoGenetics, Globe Institute, University of Copenhagen, Øster Voldgade 5-7, DK-1350 Copenhagen K, Denmark.; ^5^ Institute of Biogeochemistry and Pollutant Dynamics, ETH-Zürich, Universitätstrasse 16, 8092 Zürich, Switzerland.

## Abstract

Glacier retreat is rapidly transforming landscapes, creating thousands of new lakes that act as natural laboratories of ecological succession. In these newly formed ecosystems, colonizing animals must meet their requirements for essential organic nutrients, such as long-chain polyunsaturated fatty acids (LC-PUFAs). Here, we combined compound-specific stable isotope analyses of amino acids with fatty acid analysis to trace the origins and transfer of organic nutrients to fish (Arctic char) across a glacial-to-lacustrine lake succession in southern Greenland. Through lake succession, primary sources of nutrients shifted, coinciding with a 60% increase in LC-PUFA concentration in individual fish. In early glacial stages, Arctic char appeared to compensate for low LC-PUFA availability through endogenous biosynthesis of docosahexaenoic acid and, when connected to the fjord, by assimilating marine-derived nutrients via egg cannibalism. Overall, our results demonstrate that glacier retreat reshapes not only physical landscapes but also organic nutrient availability, with cascading consequences for animal physiology, ecosystem connectivity, and potentially long-term evolutionary trajectories.

## Teaser

As glaciers retreat in Greenland, newly formed lakes transform organic nutrient sources, increasing essential fatty acids in fish.

## INTRODUCTION

Global warming is reshaping ecosystems worldwide, with one of its most notable manifestations being the rapid retreat of glaciers across the Arctic and high mountain ranges (*1*). As glaciers retreat, they expose new landscapes that support the assembly of novel ecosystems (*2*). Notably, the number of glacial lakes formed by meltwater has increased by more than 50% over the past three decades (*3*), and in Greenland alone, approximately 6000 new lakes >10 ha are projected to form by 2100 (*4*). Newly formed glacial lakes are initially inhospitable: High turbidity limits photosynthesis (*5*) while providing relatively less protection from damaging ultraviolet radiation (*6*), and glacier flour is harmful for organisms (*7*). Over time, however, as direct glacial influence diminishes, these lakes become more suitable for life, enabling colonization by additional species (*8*–*10*), the development of complex food webs (*11*, *12*), and ultimately the potential for evolutionary processes (*13*). These lake ecosystem changes serve as natural laboratories of ecological succession, offering rare opportunities to understand how ecosystems assemble under environmental changes (*14*).

Species assembly depends not only on abiotic conditions but also on the availability of nutrients (substances needed for survival, growth, and reproduction). For example, for plants in deglaciated terrain, nitrogen often limits establishment, while associations to nitrogen-fixing bacteria facilitate early colonization (*15*). Animals (metazoa) require a wider range of organic nutrients from their food sources, termed essential nutrients. These include vitamins (*16*), some amino acids (*17*), and fatty acids (FAs) (*18*, *19*). Notably, long-chain (≥C_20_) polyunsaturated FAs (LC-PUFAs), such as docosahexaenoic acid (DHA), play a pivotal role in animal physiology (*20*–*22*). While many animals can biosynthesize LC-PUFAs from precursors (*23*), this process is energetically costly and often insufficient to meet physiological demands (*19*). While ecological succession research has largely focused on inorganic nutrient availability for primary producers (*15*, *24*, *25*), the role of organic nutrients, such as LC-PUFAs, for animals remains unexplored (*26*).

Salmonids are generally the first fish to colonize glacial lakes in the Northern Hemisphere, acting as pioneer fish species (*10*). Freshwater ecosystems typically contain less LC-PUFA than marine systems (*19*), creating the risk of nutritional limitation for colonizing fish (*27*). In glacial lakes, this constraint may be even stronger: LC-PUFA availability tends to decline with decreasing temperature (*28*), yet ectothermic consumers simultaneously face elevated LC-PUFA demands in the cold (homeoviscous adaptation) (*29*). This creates a potential mismatch: In cold glacial lakes, animal demand for LC-PUFAs is highest when environmental supply is lowest, whereas this mismatch is expected to relax as lakes disconnect from glaciers. How LC-PUFA availability shifts during glacial lake succession and how this shapes salmonid colonization and food web assembly represent an exciting avenue for exploring the role of organic nutrients in ecological succession.

In this study, we investigated the transfer of LC-PUFAs to Arctic char (i.e., char), a salmonid species, across a primary ecological succession of lakes in southern Greenland. We sampled three lake stages: (i) old lakes that have been disconnected from glaciers since the last deglaciation, 14 to 7.6 thousand years (ka) ago (*30*) (here, “old”), representing a steady-state representative of future lakes; (ii) recently disconnected lakes (10 to 150 years ago) that lost glacial influence due to contemporary warming (“recent”); and (iii) lakes that currently receives directly glacial meltwater (“glacial”). In addition, lakes varied in connectivity to the fjord, with some located above barriers that exclude migratory char, while others allowed the return of anadromous char (i.e., feeding in the fjord), potentially importing marine-derived nutrients (i.e., subsidies).

While tracing nutrient origins in natural ecosystems remains challenging, especially in the highly seasonal arctic, we combined stable isotope and FA analyses to simultaneously resolve the origins of nutrients and their trophic transfer. Specifically, we applied bulk isotopes and δ^13^C fingerprinting of essential amino acids (EAAs) to identify shifts in primary production (*31*). We hypothesized that (i) during glacial stages, constraints on primary production force food webs to rely predominantly on detrital pathways, associated with limited LC-PUFA production, and that (ii) Arctic char experience LC-PUFA limitation under these conditions, compensating through alternative sources (e.g., endogenous biosynthesis or subsidies). (iii) Total organic nutrient availability increases through lake succession in Greenland, associated with food web complexification.

## RESULTS

### Shifts in food web foundations, vertical structure, and connectivity

**Fig. 1.**
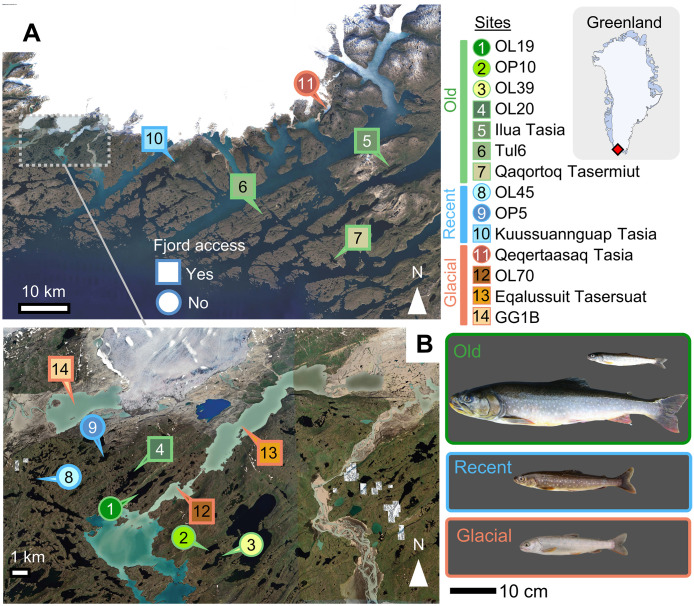
Sampling of char populations across lake stages. (**A**) Map of the study system (image: Google Earth; 2026 Airbus; US Geological Survey; Landsat/Copernicus data; IBCAO), with red diamonds indicating sampling sites in Greenland. Lake stages are color coded, and symbols denote fjord connectivity. The bottom panel shows a zoomed-in view of the area highlighted by the dashed box in the top map. (**B**) Representative adult char caught across the three lake stages.

**Fig. 2.**
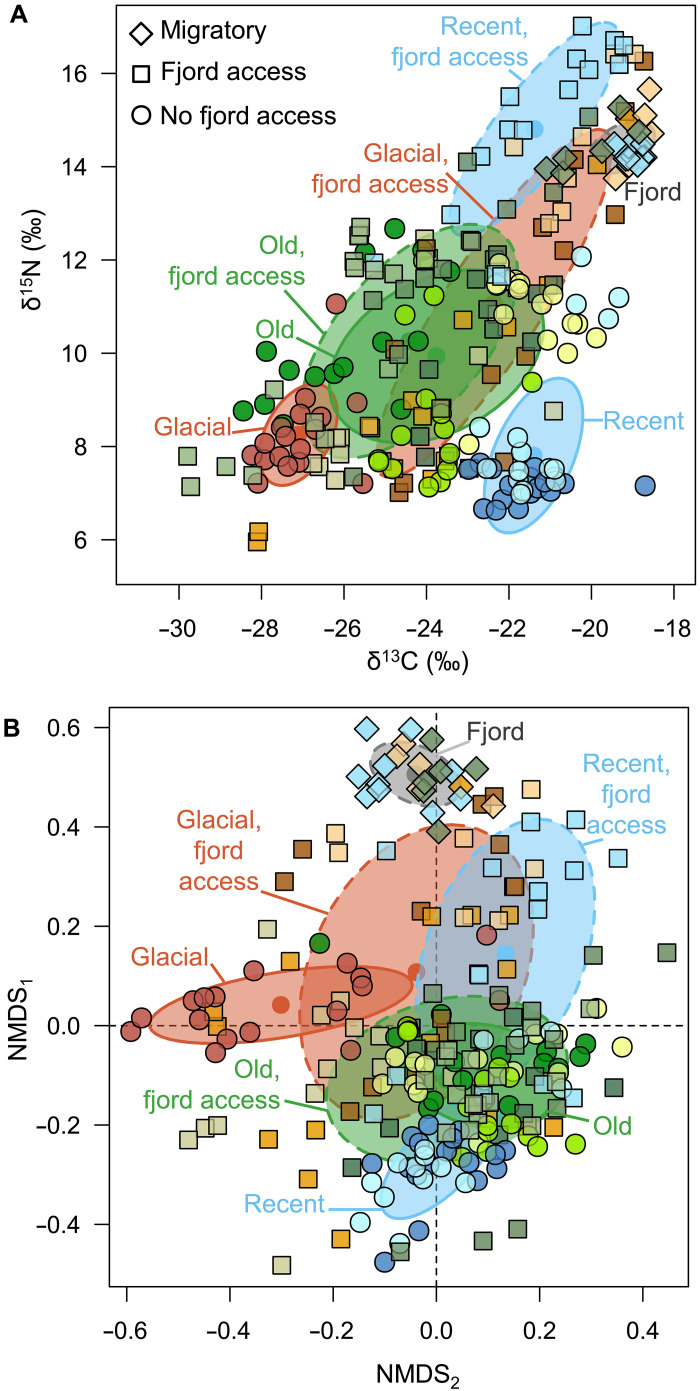
Bulk isotopes and FA profiles. (**A**) Bulk isotope biplots and (**B**) NMDS plot of FA relative abundance. Lake stages are color coded, and fjord connectivity is indicated by symbols (square, fjord access; circles, no access). Ellipses represent the 50% confidence intervals of the bimodal distribution, and lines show fjord connectivity (solid, no fjord access; dashed, fjord access). Migratory (i.e., fjord-feeding) individuals are shown with diamonds and gray ellipses. All ellipses differ significantly from one another based on pairwise PERMANOVA in both panels bulk isotopes (A) and FA relative abundance (B), except for old lakes with versus without fjord access.

We sampled char across lake successions (Fig. 1) and first investigated changes in nutrient sources and trophic dynamics. Char stomachs were dominated by chironomid larvae (78% of occurrences) and aerial invertebrates (50%), while zooplankton (17%) was rare. Piscivory, including cannibalism, was more frequent in old lakes (54% versus 10% in others; fig. S1). We found that both lake stage and fjord access changed char bulk isotope compositions and FA profiles [pairwise permutational multivariate analysis of variance (PERMANOVA), *P* < 0.05; Fig. 2, fig. S2, and table S1], except in old stages, where fjord access had no effect on either (*P* > 0.05). In lakes without fjord access, bulk δ^13^C values spanned a broad range, from the lightest mean in glacial lakes [−27.1 per mil (‰)] to the heaviest in recent lakes (−21.4‰; Fig. 2A). Migratory char showed a typical marine signature, with elevated δ^13^C (−19.3‰) and δ^15^N (14.4‰) mean values (Fig. 2A). Mixing models based on bulk isotopes indicated assimilation of marine subsidies via egg cannibalism in resident char with fjord access, with mean contributions of 37 and 73% in glacial and recent lakes, respectively (fig. S3). In contrast, although there were some individual char exceptions, marine subsidy assimilation did not differ between old lakes with and without migration (i.e., fjord connection), with all estimates overlapping zero (fig. S3). Marine subsidy assimilation was accompanied by an increase in both δ^13^C (up to −18.7‰) and δ^15^N (up to 17.0‰, the highest observed; Fig. 2A and fig. S4). This isotopic enrichment is only consistent with cannibalism on migratory char eggs, thereby supporting egg cannibalism as the primary mechanism of marine subsidy transfer. Marine subsidy reliance by egg cannibalism was also associated with increased similarity in FA profiles to migratory char (Fig. 2B), such as an increase in omega-9 gondoic acid, a marine biomarker (fig. S5).

**Fig. 3.**
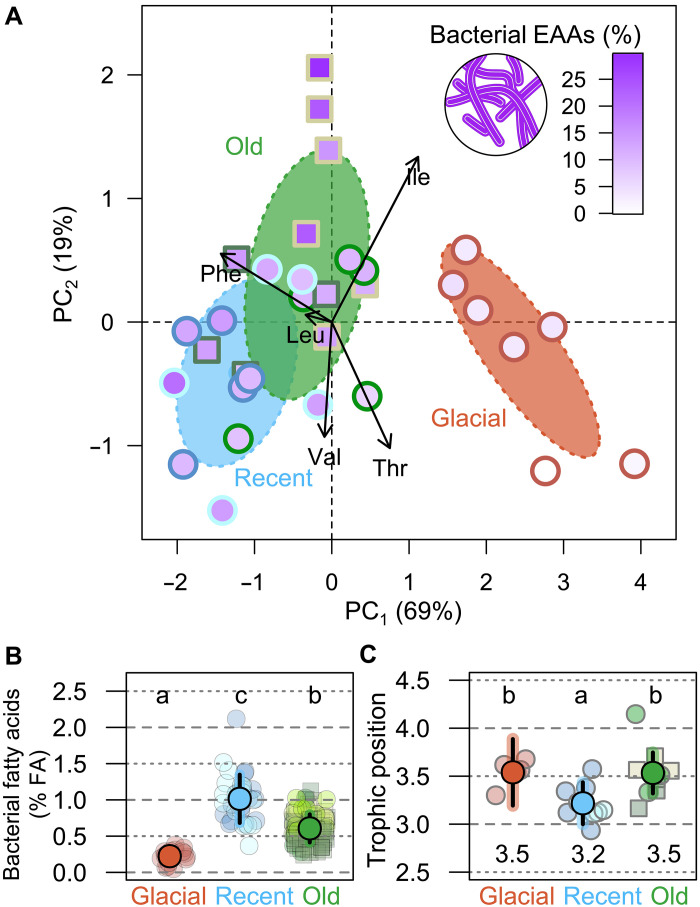
Lake food web foundation and structure. The plot shows lakes without marine subsidy reliance. (**A**) Principal components (PC) analysis of EAA δ^13^C fingerprints. Arrows indicate the loadings of the five EAAs. Each dot represents an individual char, with symbols indicating fjord connectivity (squares, access; circles, no access), inner color denotes the bacterial contribution estimated from mixing models, and outer color indicates site identity. Ellipses represent the 50% confidence distribution for each lake stage. (**B**) Relative abundance of bacterial FAs (C15:0 + C17:0; % FA). (**C**) Trophic position (TP) of small char (<20 cm). Error bars indicate 95% confidence intervals. Different letters denote significant differences among lake stages. Dot colors show lake origins.

To further understand changes within lakes, we excluded systems where char relied on marine subsidies and used compound-specific isotope analysis of amino acids (CSIA-AA) to assess the origins of primary production. Each lake stage exhibited distinct EAA δ^13^C fingerprints (pairwise PERMANOVA, all *P* < 0.05; Fig. 3A and fig. S6), indicative of shifts in primary producer communities. Bayesian mixing models indicated that EAAs were primarily of autochthonous photosynthetic origin (algae and cyanobacteria), with minimal terrestrial contribution (mean, 0.8 ± 0.1%) (fig. S7). Nonphotosynthetic bacterial sources accounted for 15 ± 5% on average (range, 5 to 30%) and were lowest in glacial lakes (mean, 8%), approximately half the contribution observed in clear-water stages (Kruskal-Wallis, *P* < 0.001; fig. S7).

The fingerprinting approach was corroborated by FA biomarkers. Terrestrial markers were nearly absent (mean, 0.09%; maximum, 0.41% FA), whereas bacterial FA markers (C15:0 + C17:0) were positively correlated with bacterial EAA contributions (*R*^2^ = 0.22, *P* < 0.01; fig. S8). Bacterial FAs were most abundant in recent lakes, intermediate in old lakes, and lowest in glacial ones, showing up to fivefold differences among stages (Fig. 3B). Mean EAA δ^13^C values confirmed that shifts in bulk δ^13^C reflected changes at the base of the food web (*R*^2^ = 0.91, *P* < 0.001; fig. S9). At the lake scale, bacterial FAs were correlated with bulk δ^13^C (*R*^2^ = 0.29, *P* < 0.001; fig. S10), indicating a link between the δ^13^C baseline and the relative importance of the detrital loop.

The δ^15^N baseline, inferred from phenylalanine (Phe) δ^15^N, did not vary significantly among lake stages (Kruskal-Wallis, *P* > 0.1; fig. S11). Consequently, trophic position (TP) estimates from CSIA-AA aligned well with bulk δ^15^N across lakes (*R*^2^ = 0.70, *P* < 0.001; fig. S12A). Char occupied a wide trophic spectrum (TP = 2.9 to 5.0), spanning three trophic levels (fig. S12). Estimates from CSIA-AA, focused on small char (<20 cm), revealed significantly lower TP in recent lakes, although the difference was modest, roughly one-third of a trophic level (Fig. 3C). Model selection showed that bulk δ^15^N values, used as a broader proxy for TP, were best explained by char standard length and lake stage (model weight > 93%; table S2), whereas models including lake size and char production received little support (model weights < 1%; table S2). In the selected model, bulk δ^15^N increased with char size (fig. S13A). After accounting for size, δ^15^N values in recent lakes were significantly lower (by 0.5 to 1.1‰) than those in old lakes, whereas no significant differences were detected between glacial and old lakes (fig. S13B). Both bulk and CSIA-AA approaches indicated similar size-corrected TPs in glacial and old lakes and lower values in recent lakes. Overall, this suite of biomarkers reflected shifts in nutrient sources (i.e., food web foundations) and transfer (i.e., vertical food web structure) across lake stages, including the assimilation of marine subsidies when lakes were connected to the fjord, which was lost in the old stage.

### Shifts in LC-PUFA availability

**Fig. 4.**
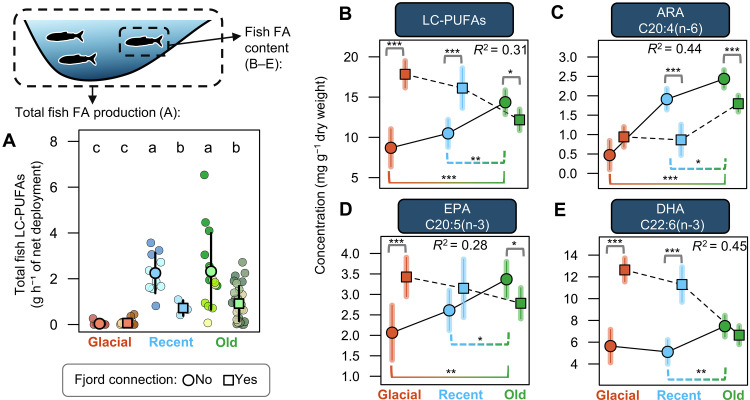
Effect of lake stages on production and amounts of LC-PUFAs in individual char. (**A**) Total production of char LC-PUFAs across lake stages. Symbols indicate fjord connectivity. Different letters indicate significant differences (pairwise Kruskal-Wallis, *P* < 0.05). h, hour. (**B** to **E**) GLM model summaries of concentration (in milligrams per gram of dry weight) of LC-PUFAs (B), ARA (C), EPA (D), and DHA (E) in char, shown as means ±1 SD. Lines connect lake stages (glacial, recently disconnected from glacier, and old clear water). Asterisks indicate significant differences between stages without fjord access and within stages comparing lakes with versus without fjord access. *P < 0.05, **P < 0.01, ***P < 0.001.

Across lake stages, we observed pronounced differences in total LC-PUFA fish production, expressed as total resident fish LC-PUFA standardized per unit effort. Glacial lakes contained over 60 times less fish LC-PUFA than clear-water stages (recent and old lakes), while recent and old stages did not differ significantly (Fig. 4A). Fjord access had no effect on total LC-PUFA accumulation in glacial lakes but reduced total LC-PUFA by approximately two- to threefold in clear-water stages (Fig. 4A). Those effects were mainly driven by overall changes in fish production (fig. S14).

In char, LC-PUFAs were dominated by the omega-3 DHA (56%), eicosapentaenoic acid (EPA; 22%), and the omega-6 arachidonic acid (ARA; 13%). Model selection showed that concentration in fish (in milligrams per gram of dry weight, reflecting total nutrient accumulation in tissue) for total LC-PUFAs, ARA, EPA, and DHA was best explained by an interaction between lake stage and fjord access, with an additional effect of fish size on LC-PUFA, EPA, and DHA concentrations (table S3). Models differing in the treatment of fish size, either excluding it or including it as an interaction with stage and anadromy, had similar support, whereas all remaining models received little support (<2%; table S3). In the selected top-ranked models, the effect of size was always negative (fig. S15), although not significant for DHA. Without fjord access, LC-PUFA concentration increased from glacial to old stages (+64%, *P* < 0.001; Fig. 4B) and from recent to old stages (+37%, *P* < 0.01; Fig. 4B), driven mainly by rises in ARA (Fig. 4C) and EPA (Fig. 4D). DHA concentrations differed significantly only between recent and old stages (+46%, *P* < 0.01; Fig. 4E).

Fjord access strongly influenced LC-PUFA concentration in glacial and recent stages, with the largest effect in glacial lakes, where LC-PUFA concentration doubled, reaching the highest observed values (∼18 mg g^−1^; Fig. 4B). This increase was primarily driven by DHA (Fig. 4E), with moderate contributions from EPA (Fig. 4D).

### Processes controlling DHA relative abundance in char

To understand the dietary and metabolic origins of DHA, we examined changes in its relative abundance (% FA), as this metric better reflects compositional variation in FAs from underlying sources, independent of variation in total lipid concentration among individuals. Across all lake stages, some individual char exhibited low DHA (<20% FA; fig. S2). Consistently, DHA and stearidonic acid (SDA) (precursor involved in DHA synthesis) were strongly negatively correlated (*P* < 0.001 across all stages; fig. S16), supporting SDA accumulation linked to endogenous DHA synthesis. In glacial lakes, SDA was particularly elevated in some individuals (∼10% FA; fig. S2) and was tightly related to DHA (*R*^2^ = 0.91; fig. S16). In contrast, SDA (% FA) showed no relationship with dietary source indicators, including essential EAA δ^13^C fingerprinting, δ^13^C of EAAs, or bulk δ^13^C (fig. S17), indicating an endogenous rather than dietary origin.

**Fig. 5.**
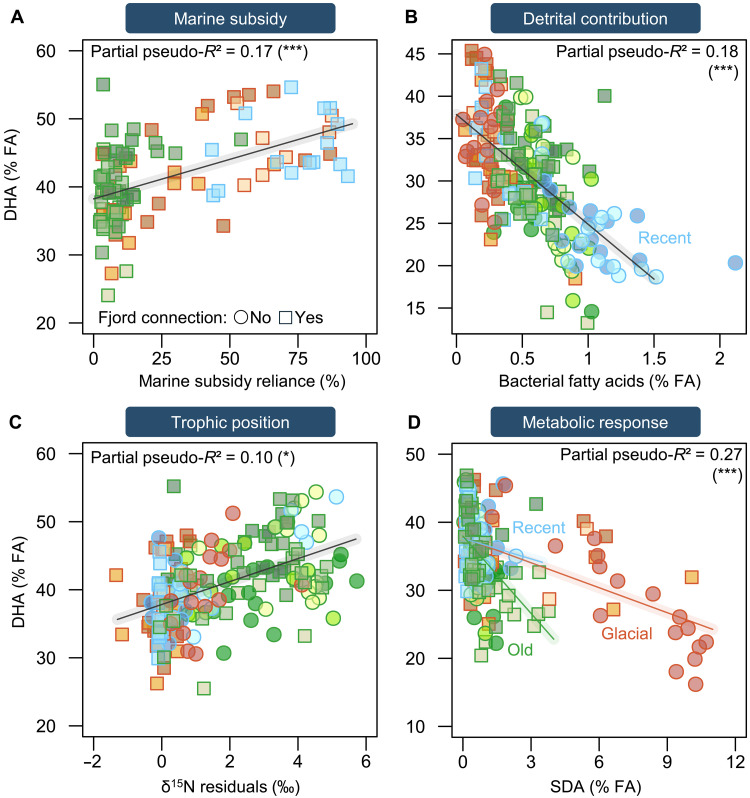
Origins of DHA in char. Partial residual plots of DHA relative abundance (% FA) as a function of marine subsidy reliance via egg cannibalism (**A**); detrital contribution, inferred from bacterial FAs (% FA) (**B**); relative TP change in fresh water, inferred from δ^15^N residuals (after accounting for marine reliance) (**C**); and metabolic response, indicated by SDA (% FA), and its interactive effect with lake stage (**D**). Relationships are shown after accounting for other variables in the model. Lines indicate linear regressions for visualization of model effects, with associated partial pseudo-*R*^2^ and significance (*P < 0.05 and ***P < 0.001). Each point represents an individual char; symbols indicate fjord connectivity (squares, access; circles, no access), inner colors indicate lake origin (Fig. 1), and outer colors indicate lake stage (see legend).

Model selection identified a top-supported model (weight = 76%) including six predictors, as well as an alternative model that additionally included the interaction between char size and lake stage; all other models received little support (model weight < 1%; table S4). The selected model explained 66% of the variation in DHA (% FA) and included additive effects of marine subsidy reliance (Fig. 5A), detrital reliance (Fig. 5B), TP (Fig. 5C), and body size (negative but not significant; *P* > 0.05), as well as an interaction between lake stage and metabolic regulation, as indicated by SDA (% FA) (Fig. 5D). Notably, this interaction shows that char in glacial lakes exhibited significantly higher SDA at similarly low DHA (% FA) compared to those in old lakes (Fig. 5D).

Together, 66% of the variation in DHA (% FA) was explained by changes in food web foundations, particularly the balance between marine and freshwater reliance (Fig. 5A) and the importance of detrital pathways within fresh water (Fig. 5B), changes in food web vertical structure (char TP; Fig. 5C), and endogenous biosynthesis (Fig. 5D), as indicated by elevated SDA when DHA was low, especially in glacial stages without fjord access.

## DISCUSSION

**Fig. 6.**
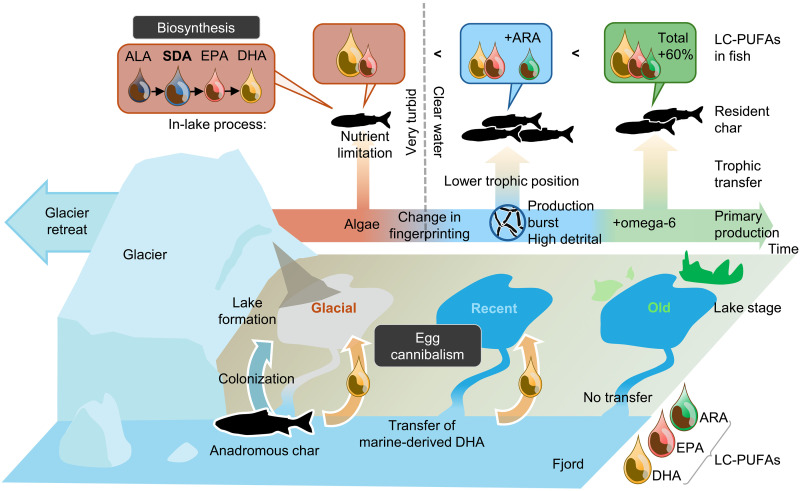
Summary. Conceptual evolution of lakes following glacier retreat into glacial, recent, and old stages, with consequences for lake LC-PUFA production, fish regulation, and the transfer of marine-derived organic nutrients.

Our study reveals that lake ecological succession following glacial retreat in Greenland reshapes food web foundations, particularly the dependence to marine and detrital production, accompanied by marked changes in organic nutrient availability to animals (Fig. 6). These findings provide rare empirical evidence for the role of organic nutrients in ecological succession, highlighting that environmental change creates new nutritional landscapes with cascading ecological and evolutionary effects.

### Ecological succession from glacial to clear-water lakes

We report established resident fish populations in an ice-contact glacial lake under extreme conditions (euphotic zone < 0.5 m), raising questions about the foundations (i.e., source of basal production) of these food webs. Contrary to expectations that glacial flour sustains detrital pathways (*32*), our EAA fingerprinting revealed that algae mainly fuels fish production, with distinct fingerprinting (relatively low Phe δ^13^C) of glacially adapted algae.

Food web biomarkers change as lakes get disconnected from glaciers. Recent clear-water lakes showed peaks in bacterial markers, increasing baseline δ^13^C values [more complete utilization of carbon in primary production (*33*)], and lower fish TP, signaling a “burst” of productivity efficiently transferred to fish. This burst is likely fueled by the mobilization of glacier-derived organic carbon (*32*) and glacier-derived nutrients [e.g., phosphorus (*34*) and iron (*35*)], coupled to enhanced growth conditions for algae (*5*) and invertebrates (*7*). Benthic pathways may channelize this burst (*36*), as supported by the absence of zooplankton in char stomachs from these recent lakes.

With transition to clear water, bacterial contributions diminished, and the transient production burst faded, giving rise to more conventional lacustrine food webs with intermediate detrital support (*37*). In parallel, omega-6 FAs started to accumulate in fish, suggesting new sources of production, likely from bacteria and macrophytes (*37*), although they contributed little to overall protein sources. While glaciers are a source of nitrogen (*38*), baseline δ^15^N values remained stable, suggesting that this proxy poorly reflects changes in nitrogen sources or that glacial-derived nitrogen is made available to primary producers to a limited extent.

Although old lakes contained more large, high-TP individuals, likely due to the presence of sticklebacks (supported by stomach content), the TP for a given char size was similar between glacial and old lakes, as indicated by both CSIA-AA and bulk δ^15^N values. In contrast, TP was lower in recent lakes and showed no relationship with lake size or total production. Instead, we suggest that the increased productivity (i.e., the burst described above) reduces size-corrected fish TP, consistent with the idea that more productive systems support higher transfer efficiency to consumers (i.e., less intermediates) (*39*). However, this pattern may be specific to early stages of lake ontogeny and may not hold in larger, more complex lakes hosting more fish species, where additional trophic levels could be added (*40*).

Last, lake succession is likely to differ substantially across regions. Glacial disconnection is often accompanied by the expansion of surrounding vegetation and the release of dissolved terrestrial carbon (i.e., lake browning), particularly in North America (*24*) and the Eurasian Arctic (*25*). In contrast, Greenland lakes receive much lower terrestrial inputs (*41*), although shifts under atmospheric and climatic conditions can modify these patterns (*42*). In our focal system, the use of multiple biomarkers illustrates how the food web architecture (primary production and vertical structure) shifts as lakes lose glacial influence, providing an example of ecological succession in action.

### Increasing organic nutrient availability for animals in ecological succession

An open question in ecological succession is how organic nutrient availability, such as LC-PUFAs, influences the establishment of higher trophic levels (*19*, *26*). We found that total LC-PUFAs, and particularly EPA, declined with increasing char size, consistent with previous findings (*43*). This reflects the mobilization of organic nutrients toward reproduction in larger individuals, whereas LC-PUFAs are preferentially retained to support growth and development in juvenile fish (*44*).

Total fish LC-PUFA accumulation increased by nearly two orders of magnitude as lakes transitioned to clear water, reflecting the negative effect of glacial influence on algal production (*5*, *6*), the primary source of LC-PUFAs (*19*). LC-PUFA preservation is favored by reduced oxidation under low temperatures and high turbidity (*45*), but these conditions seem insufficient to offset reductions in overall production. While LC-PUFA requirements are expected to be highest during the glacial stage (*29*), evidence for nutritional limitation is further supported by an ∼60% increase in LC-PUFA concentration, after accounting for fish size, in char across the succession. This increase arises from a two-step process. First, as lakes clear, char integrate new LC-PUFA sources, notably omega-6 ARA, another metabolically important nutrient (*46*). Second, in older lake stages, LC-PUFA enrichment reflects declining detrital inputs, consistent with the fact that algae provides higher nutritional quality than bacterial sources (*47*).

Despite pronounced differences in LC-PUFA concentration in char among lakes, DHA concentration in char remained similar between glacial and old stages. DHA is particularly important for high-level consumers, playing a key role in neural and sensory tissues (*48*, *49*). We found that DHA (% FA), reflecting its compositional abundance within the FA pool, increased with TP, reflecting its preferential bioaccumulation along food chains (*50*, *51*). This may counteract the negative effect of body size on LC-PUFA accumulation, so that DHA concentration remains stable as char occupy higher TPs with increasing size. In addition, fish are able to compensate for low DHA availability by biosynthesis based on elongation and desaturation of FAs (*23*, *27*). We found that fish with low DHA (% FA) had higher SDA (% FA), a precursor in DHA biosynthesis (*52*). Our diet biomarkers supported the endogenous origin of SDA, and so of DHA biosynthesis, but only analysis of gene expression could confirm those findings. Char in glacial lakes showed the strongest metabolic response: For similarly low DHA, they accumulated more SDA. This could be due either to gene up-regulation of α-linolenic acid (ALA) desaturation into SDA (*53*) following homeoviscous adaptation (*29*), and inefficient conversion of SDA by elongase (Evolv5), especially at colder temperatures (*54*). In both cases, the change in metabolic response suggests greater nutritional stress in glacial environments.

Our results provide empirical evidence that organic nutrient availability shifts across ecological succession. The idea that nutrient availability can influence species assembly resonates across ecosystems. For example, in coastal California, the nutritional quality of sea urchins determines sea otter distributions, driving top-down trophic cascades that govern the shift between barren grounds into kelp forests (*55*). In lakes, LC-PUFA availability may influence a range of consumers, including invertebrates (*18*), other fish (*21*, *27*, *53*), and riparian predators (*20*), potentially altering their trophic interactions and, in turn, species assembly across the succession. While our results reveal broad patterns of organic nutrient evolution linked to primary production, incorporating lower trophic levels would further illuminate the mechanisms of food web complexification (*56*).

### Effect of organic nutrient availability on nutrient fluxes between ecosystems

Char can migrate into fjords for foraging (i.e., anadromy), a strategy shaped by a trade-off between predation risk and foraging opportunities (*57*). The anadromous migrations of salmonids are widely recognized as vectors of marine subsidies to inland ecosystems, delivering nutrients (i.e., subsidies), through dissolved inputs, carcasses, and eggs (*58*), with far-reaching ecological consequences (*59*). Here, we provide clear evidence of this marine subsidy transfer driven by cannibalism on anadromous char eggs, only in glacial and recent lakes. Egg cannibalism, a common behavior in char (*60*), provide a food source rich in DHA and may therefore facilitate the persistence of char as the only pioneering fish species in nascent glacial lakes where other species (e.g., threespine stickelback, *Gasterosteus aculeatus*) cannot yet establish.

The decline in marine-derived LC-PUFA transfer as lakes lose glacial connectivity aligns with the theory that as lake nutrient availability improves, the relative importance of subsidies diminishes (*60*). Moreover, the ecosystem-scale consequences of subsidies are not additive for LC-PUFA total production, as char migration either had no effect (glacial lakes) or substantially reduced total char LC-PUFAs (clear-water stages). This pattern likely reflects multiple mechanisms. Juvenile out-migration can act as a biomass export (*61*), while anadromous char spawning elevates juvenile densities, increasing juvenile competition (*62*) and potentially cascading to affect primary production (*63*).

These findings resonate with broader concerns about world production of LC-PUFAs (*64*), where rising human demand coincides with declining natural production (*65*). Salmonids, as high-value fisheries resources, are an important source of LC-PUFAs (*66*). Glacier retreat generates new habitats that could initially support salmonid expansion (*10*). However, as glaciers continue to disconnect, the loss of migration may be favored as lake nutritional quality improves, creating a positive feedback that further enhances nutrient accumulation and, in turn, further reduces the production of anadromous salmonids. These dynamics are further complicated by the simultaneous impacts of global change and glacier retreat on fjord ecosystems (*35*), as well as surrounding vegetation and fluxes of terrestrial carbon to rivers and lakes (*24*, *42*). Anticipating how glacier retreat will ultimately shape salmonid LC-PUFA production requires integrating environmental change at the landscape level (*67*).

### Impact of organic nutrient availability on evolutionary potential

The ancestral form of char is migratory, raising a central question: How do char adapt to the lower availability of LC-PUFAs in fresh water compared to marine environments (*27*)? Evolution of genes encoding LC-PUFA biosynthetic enzymes represents a critical step in marine-to-freshwater transitions, as shown in flatfish (*68*) and polychaetes (*69*). Likewise, increased copy numbers of Fads2 (a desaturase involved in DHA endosynthesis) are associated with freshwater colonization of sticklebacks (*27*). However, our data reveal that char from old lakes show high concentration of LC-PUFAs, similar to anadromous ones, suggesting that nutritional constraints relax over time. This pattern echoes findings in sticklebacks from old lakes in southern Greenland, where Fads2 copy numbers are similar between marine and lake populations (*53*).

Over evolutionary timescales, the emergence of new dietary niches following glacier retreat can promote adaptive radiation, as seen in char in Greenland (*13*). This diversification coincides with high LC-PUFA availability, raising questions on the potential role of nutritional resources in shaping evolutionary trajectories (*26*). LC-PUFAs are critical not only for somatic growth (*21*) but also for cognitive development (*22*), implying that shifts in the nutrient availability may influence evolutionary potential. For instance, enhanced DHA availability could initially facilitate the loss of anadromy and later enable the evolution of alternative life history strategies, such as piscivory, since DHA is a critical nutrient for predator performance (*70*).

Although glacier retreat has local positive effects for ecosystems, as observed in our study, these localized gains do not compensate for the worldwide accelerating global losses in biodiversity (*71*). Still, environmental change offers opportunities to study ecological succession in real time. Overall, our study underscores the importance of considering organic nutrients, such as LC-PUFAs, in understanding ecosystem succession, both across short timescales (nutrient constraint and ecosystem connectivity) and longer ones (adaptive potential).

## MATERIALS AND METHODS

### Study sites

We conducted our study across 14 lakes selected to represent different stages of ecological succession. Lake geological histories were inferred from published literature, we provide details in table S5. The lakes were primarily deglaciated between 14 and 7.9 ka and became fresh water following isolation from the sea at ∼7.6 ka or earlier. We categorized lakes into three stages based on their current and historical connectivity to glaciers: proglacial lakes directly fed by glaciers (*n* = 4; hereafter glacial), lakes that have recently lost direct glacial inflow (*n* = 3; hereafter recent), and clear-water lakes that have remained hydrologically disconnected from glaciers for an extended period (*n* = 7; hereafter old). The recent category includes lakes that became disconnected from glaciers due to modern warming and glacier retreat following the Little Ice Age (*72*). To further determine the timing of glacier-lake disconnection, we used Google Earth to visually identify the transition from glacial to nonglacial conditions, which is marked by a distinct color change (from brown to dark blue; fig. S18). One lake (OP5) already appeared nonglacial in the earliest available satellite imagery (1985), indicating that the transition likely occurred sometime between the end of the Little Ice Age and 1985.

We sampled lakes in July to August 2024 and one lake in July 2022 (Qaqortoq Tasermiut). We measured lake surface area using satellite imagery (Google Earth), recorded in the field maximum depth using sonar, and measured Secchi depth. Lake area ranged from 11 to 628 ha (mean, 137 ha), and maximum depths ranged from 6 to 95 m (mean, 29 m).

Secchi depths ranged from 10 cm to 10 m and were significantly different between glacial lakes (mean, 0.15 m; range, 0.1 to 0.2 m) and following lake stages (mean, 6.4 m; range, 4.5 to 10 m) (*P* = 0.01, Kruskal-Wallis test). There was no significant difference between recent and old stages (*P* > 0.05); thus, we classified both as clear-water stages. All data can be found in table S5.

### Fish collection

We sampled fish using standardized overnight deployments of multimesh gillnets (details in text S1). Fish communities were composed of char (*Salvelinus alpinus*), our focal species, and, in clear-water lakes, we also captured threespine stickleback (*G. aculeatus*), hereby stickleback. After capture, we euthanized the fish with an overdose of clove oil. We photographed each individual char, measured standard and total lengths to the nearest millimeters, weighed them to the nearest 0.1 g, and collected a muscle biopsy from the dorsal region for analyses (see below). We fixed the fish in formalin in the field and then transported them to Eawag, Kastanienbaum, Switzerland, where fish were washed and archived.

We estimated catch per unit effort (CPUE) as the number and biomass of char captured per net, standardized by the duration the net was deployed. We estimated stickleback weights based on FL (fork length) measurements and log-transformed weight-FL relationships from a subset. The biomass of sticklebacks accounted for less than 2.5% of the total resident fish biomass across all lakes (average, 0.4%), supporting that focusing on char provided a representative estimate of total fish biomass and nutrient accumulation.

We categorized the lakes based on their connectivity to the fjord, which enables seasonal migration of char to feed in the fjord (anadromy). Migration was confirmed in some lakes by the capture of anadromous individuals, identified by distinct phenotypic traits such as a short head, reduced fins, larger body size, and silver coloration (*60*). These visual assessments were further validated through stable isotope and FA analyses (see Results). In other lakes, while anadromous individuals were not captured, local fishers reported seasonal migrations to the fjord. Lakes with physical barriers, such as large waterfalls that prevent return migration, were identified through field observations and used as controls representing nonmigratory systems.

Char diets in clear-water Greenland lakes typically include benthic invertebrates, zooplankton, and sticklebacks (*13*). To provide further insights, we analyzed stomach contents for a subset of individuals (text S2).

### Fatty acids

Muscle tissue typically reflects dietary nutrients from the past few months of growth, thereby particularly relevant in highly seasonal Arctic systems (*73*), integrating roughly the annual growth period (*74*). It therefore captures longer-term nutrient acquisition that can be linked to primary production using biomarkers (see below). We analyzed bulk isotopes and FA abundance from dorsal muscle biopsies of 222 char. We analyzed 22 anadromous char and selected a subset of 200 resident individuals. To do so, we randomly selected ∼15 fish per lake spanning the observed size range or included all individuals when fewer than 15 were available (fig. S19).

We analyzed FAs using gas chromatography–flame ionization detection following a previous protocol (*51*); more information about sample preservation and analysis can be found in text S3. We report FA concentration relative to muscle dry weight (in milligrams per gram of dry weight, hereafter “concentration”) and FA relative abundance as the percentage of total FAs (% FA). FA concentration reflects the amount accumulated in fish tissues and is therefore more closely related to FA availability (see the “LC-PUFA accumulation in char” section). In contrast, FA relative abundance captures compositional variation and is more informative about differences in FA sources and production pathways (*75*) (see the “Changes in fish food source: Bulk isotopes and FA profiles,” “Changes in food web foundation: EAA δ^13^C fingerprinting and FA biomarkers,” and “Origins of DHA in char” sections). We categorized FAs into saturated FAs (SAFAs), monounsaturated FAs, and PUFAs and further subdivided PUFAs into short-chain PUFAs (≤C_20_) and LC-PUFAs (≥C_20_).

### Bulk and amino acid stable isotopes

We performed bulk isotope analyses on lipid-extracted samples; more information can be found in text S4. We ran CSIA-AA for a subset of fish (*n* = 39). We selected individual fish to represent the main lake archetypes identified in the study based on bulk isotopes and FAs (see Results). Our selection included two glacial lakes, one with fjord access (Qeqertaasaq Tasia) and one without (Eqalussuit Tasersuat), two recent clear-water lakes (OP5 and OL45), and three old clear-water lakes (OL19, OL20, and QL1).

We derivatized the amino acids to *N*-acetyl methyl ester derivatives following previous protocols (*31*, *76*) and measured isotopic ratios on gas chromatography–combustion–isotope ratio mass spectrometry; more information can be found in text S5. Because glutamine is deaminated during the hydrolysis to glutamic acid, here, we report isotopic composition for Glx (glutamic acid + glutamine).

### Permits

This research was done under research permits G22-057 and G24-057 from the Naalakkersuisut (Government of Greenland).

### Statistical analyses

All statistical analyses were done in R (*77*).

### Changes in fish food source: Bulk isotopes and FA profiles

To infer shifts in the sources and transfer pathways of nutrients, we analyzed bulk stable isotopes (δ^13^C and δ^15^N) and FA relative abundance (% FA) as indicators of dietary differences. The total composition of FAs (% FA) is hereafter referred to as “FA profiles.” FAs with low concentration (maximum, <0.5 mg g^−1^), and those for which more than 90% of fish had zero values were excluded from the analysis.

To assess the effects of lake stage and fjord access (including fjord-feeding individuals as a distinct category), we conducted a PERMANOVA on both bulk isotopic values and FA profiles with the *vegan* package (*78*). We used pairwise PERMANOVA to test for differences between each group, with *P* values adjusted using the Holm correction. We used Euclidean distance for bulk isotopes and Bray-Curtis distance for FAs. We visualized differences in FA profiles among char categories using non-metric multidimensional scaling (NMDS). We drew ellipses representing bivariate normal distributions using the *car* package (*79*).

Last, we tested each individual FA (% FA) for differences across the seven categories using Kruskal-Wallis tests, which we adjusted for multiple comparisons using the Holm method with the *agricolae* package (*80*).

### Reliance on marine subsidies via egg cannibalism

A previous study showed that char in Greenland can rely on marine subsidies through cannibalism on anadromous char eggs, which acts as a primary mechanism for the transfer of marine-derived nutrients, rather than other pathways such as carcass scavenging (*60*). We quantified marine subsidy reliance via egg cannibalism by implementing a mixing model in the package *MixSIAR* (*81*), using resident char and migratory adult char as integrated end-members for fresh water and marine sources, respectively, and bulk isotopes as dietary markers (*60*); more details can be found in text S6. We compared marine resource utilization using Holm-adjusted Kruskal-Wallis tests. We tested the relationship between reliance on marine subsidies and the transfer of C20:1n-9, a marker of Arctic marine pelagic food chains (*75*).

### Changes in food web foundation: EAA δ^13^C fingerprinting and FA biomarkers

We aimed to determine whether lake stages influence the foundations of food webs (i.e., origins of organic nutrients), particularly the relative importance of algal production compared to the detrital loop (bacterial production). These shifts in primary production, which support higher consumers, can be tracked because primary producers differ in their synthesis of molecules, including FAs and EAAs. Here, we combined EAA δ^13^C fingerprinting with FA biomarkers. Because we found evidence of high levels of marine subsidy reliance in glacial and recent lakes with fjord access and in some char in one old lake, Ilua Tasia, although to a lesser extent (see Results), we excluded those lakes to focus on in-lake freshwater processes only.

We first applied the EAA δ^13^C fingerprinting. This approach is particularly valuable because EAAs are ubiquitous but synthesized only by primary producers, with little-to-no isotopic fractionation along food chains (*82*). In addition, the relative offsets between EAA δ^13^C values reflect differences in anabolic pathways (*31*, *83*), largely independent of environmental conditions (*31*), a pattern referred to as “fingerprinting.” Terrestrial plants, algae, and bacteria display distinct δ^13^C fingerprints (*31*, *76*), allowing us to estimate the contribution of each production source to higher consumers (*82*). We implemented a mixing model in the package *MixSIAR* (*81*) using literature data of algae (including cyanobacteria), bacteria, and plants; more details can be found in text S7.

Second, we used FA markers as a qualitative complementary approach (*37*, *75*). We focused the analysis on SAFAs because they are less metabolically regulated in consumers (*84*). Bacteria produce distinctive FAs, including odd-chain FAs and isobranched FAs (*75*). Here, we used the sum of C15:0 and C17:0 (% FA), as they are bacterial biomarkers and abundant in fish (*85*). We did not use isobranched odd-chain FAs as bacterial biomarkers because of analytical challenges, as they were present in much lower quantities (more than two orders of magnitude less). We used the sum of C20:0, C22:0, and C24:0 (% FA) as markers of terrestrial plant input in fish (*37*, *47*). We fitted linear models relating these FA markers to the EAA fingerprinting model results.

### Trophic position

We used Phe to infer a baseline for δ^15^N in primary production (δ^15^N_Phe_) and calculated fish TP using the CSIA-AA method based on the relative δ^15^N enrichment of Glx (trophic amino acid); details are provided in text S8 (*86*, *87*). We tested whether char TP differed among lake stages using two complementary analyses. To avoid confounding effects from marine subsidies, we restricted the analysis to lakes without marine subsidy reliance. First, we compared char TP estimated from CSIA-AA, restricting this analysis to individuals with a standard length (SL) of <20 cm, which represent the most abundant size class (72% of the resident catch) and allowed comparisons with small sample size across lakes while minimizing size-related variation. Differences among stages were tested using pairwise Welch’s modified two-sample *t* tests based on sample size, mean TP, and individual TP SD. Second, we examined broader patterns in char TP using bulk δ^15^N as a proxy. We first evaluated the relationship between CSIA-AA–derived TP estimates and bulk δ^15^N values. Bulk δ^15^N was then analyzed using a linear mixed-effects model implemented in the *lme4* package (*88*), with lake included as a random effect to account for baseline isotopic differences among lakes. Fixed effects included fish SL, CPUE (as a proxy for fish production), lake size, lake stage, and their pairwise interactions. We performed a model selection using the Akaike information criterion (AIC), implemented with the dredge function from the *MuMIn* package (*89*), and conducted post hoc comparisons with Holm adjustment using the *emmeans* package (*90*).

### LC-PUFA accumulation in char

To assess the total accumulation of LC-PUFAs in char across lakes, we quantified total fish LC-PUFAs standardized by unit effort. As char catches drop significantly below 25 m and because some lakes are shallow, we restricted our analysis to nets deployed in the upper water column (<25 m). CPUE was defined as the duration of gillnet deployment in each lake (in hours). The total LC-PUFA yield per hour of netting was calculated by multiplying char biomass CPUE by the lake mean LC-PUFA concentration. To compare LC-PUFA accumulation across lake categories (glacial stage and fjord access, six categories), we used a nonparametric pairwise comparison Kruskal-Wallis test, which we adjusted for multiple testing using the Holm method in the *agricolae* package (*80*).

To test whether char accumulated different concentration of LC-PUFAs, we fitted generalized linear models (GLMs) for total LC-PUFAs and for each of the three main LC-PUFA individually: ARA, EPA, and DHA. We included as potential predictors lake glacial stage, fjord access, fish length, and their one-way interactions. We included fish length as a factor, as fish regulate FA composition differently across ontogeny and, more generally, with size (*43*). We did not include lake identity as a random effect, as some lake categories were represented by only a single site, preventing reliable estimation of between-lake variance. We performed a model selection using the AIC (*88*). We computed effect sizes using the *effects* package (*91*) and conducted post hoc comparisons with Holm adjustment using the *emmeans* package (*90*). We computed pseudo-*R*^2^ using the Cox-Snell index with Nagelkerke correction, implemented with the function pR2() from the *pscl* package (*92*).

### Origins of DHA in char

We investigated the origins of DHA in char by focusing on DHA relative abundance (% FA), as this metric reflects source contributions more directly than total intake or accumulation. First, we examined whether DHA (% FA) was related to endogenous synthesis. In fish, the biosynthesis of DHA proceeds through a series of elongation and desaturation steps (*27*, *93*), from ALA (18:3n-3) to SDA (18:4n-3), EPA (20:5n-3), docosapentaenoic acid (22:5n-3), and lastly to DHA (22:6n-3). To assess whether DHA (% FA) was linked to enhanced synthesis of its precursors, we computed Pearson correlation matrices among these PUFAs using the *psych* package (*94*). Because SDA was consistently and strongly correlated with DHA across lakes (see Results), we included SDA as a potential variable of metabolic regulation of DHA.

Second, we implemented a GLM of DHA (% FA), including as potential predictors marine subsidy reliance, detrital reliance, TP, body size, and metabolic response (SDA), as well as interactions between stage and both size and SDA, to test whether metabolic responses were stage dependent. Marine subsidy reliance was derived from the bulk mixing model; values were set to zero for all char in lakes without fjord access, as these represented false contributions (*n* = 7 of 93 individuals had values strictly >1%). Detrital reliance was derived from bacterial FAs (C15:0 + C17:0). TP was corrected for the increase in δ^15^N associated with marine subsidy reliance using the residuals of this relationship (fig. S4B), thereby representing relative variation in δ^15^N within freshwater systems. Lake size can influence FA profiles by changing the relative importance of benthic production (*37*). However, we did not include this factor here, as our goal was to directly link DHA (% FA) variability to changes in nutrient sources (e.g., detrital reliance). Model selection and effect computation followed the approach described above (see the “LC-PUFA accumulation in char” section).

## Supplementary Materials

### The PDF file includes:

Supplementary Text Figs. S1 to S19 Legends for tables S1 to S5 References
