# Supplementary material for: Glacier retreat shapes organic nutrient availability across lake succession
Source: Sci Adv. 2026 Aug 7;12(32):eaef3201. doi: 10.1126/sciadv.aef3201 (PMC13450304; doi:10.1126/sciadv.aef3201)
Supplement: Supplementary file 1 [file sciadv.aef3201_sm.pdf]

Science Advances
AAAS

Supplementary Materials for

Glacier retreat shapes organic nutrient availability across lake succession

Grégoire Saboret *et al.*

Corresponding author: Grégoire Saboret, gsaboret@ucsc.edu

Sci. Adv. **12**, eaef3201 (2026)
DOI: 10.1126/sciadv.aef3201

The PDF file includes:

Supplementary Text
Figs. S1 to S19
Legends for tables S1 to S5
References

Other Supplementary Material for this manuscript includes the following:

Tables S1 to S5

SUPPLEMENTARY TEXT

Supplemental Text 1: Fish sampling

Multi-mesh gillnets were set at various depths in the lakes, spanning the gradient from near shore (1–3 m) to the deepest points (mean depth 13 m, range 1–93 m). Each net measured 30 m in length and 1.5 m in height and included a range of mesh sizes (knot-to-knot) according to the Central European standard protocol for gillnet fishing: 5.0, 6.25, 8.0, 10.0, 12.5, 15.5, 19.5, 24.0, 29.0, 35.0, 43.0, and 55 mm. Nets were typically left overnight (mean 21 hours), except in lakes with high fish abundance (minimum 3 hours) or in glacial lakes with low abundance, where nets were left longer (maximum 47 hours).

Supplemental Text 2: Stomach contents

We analyzed the stomach contents of a subset of formalin-preserved char. The subset was randomly selected to include approximately 10 individuals per lake; however, some fish could not be analyzed due to tissue degradation from high formalin concentrations. This selection was also different from the selection for FA/bulk isotope analysis. In total, 68 char from nine lakes were examined: glacial (n = 4), recent (n = 3), and old (n = 2). Stomachs were opened and screened for identifiable remains, which were categorized as chironomid larvae, aerial invertebrates (e.g., Diptera in the aerial phase), other zoobenthos, zooplankton, stickleback, char, unidentified fish (i.e., char or stickleback), and char eggs. The presence or absence of each category was recorded.
Changes in the presence of prey categories across lake stages were analyzed using pairwise chi-squared tests with Holm correction for multiple comparisons.

Supplemental Text 3: Fatty acid analysis

We preserved all samples at –20 °C for 5 to 9 months before freeze-drying and analysis. For one lake (QL1), we stored the samples at –20 °C for approximately three years. Although long-term storage at –20 °C may lead to degradation of long-chain polyunsaturated fatty acids (LC-PUFAs), with storage at –80 °C being recommended (*100*), previous studies have shown that the effects of storage temperature vary across sample types and tend to be weaker in fish muscle, with no changes in fatty acid (FA) profiles and abundance at -20°C/-30°C for 1-10 years (*100*). Most importantly, maintaining stable, low-oxygen storage conditions is critical (*100*). We kept all samples at –20 °C in sealed tubes continuously from collection until analysis. Therefore, we assumed that our dataset reliably represented the natural FA composition, despite the possibility of some short-term post-capture degradation, which would however be comparable across sites.

We freeze-dried the muscle samples for 48 hours and homogenized them with a spatula. We weighed an average of 5 mg of dried tissue per sample, which we used for fatty acid, bulk, and amino acid isotope analyses. We added 23:0 methyl ester (ME) as an internal standard. To extract fatty acids, we performed two successive extractions using dichloromethane/methanol (2:1 v/v). For the first extraction, we added 4 mL of solvent, sonicated the samples for 15 minutes in an ultrasonic bath, and incubated them overnight. For

the second extraction, we used 2 mL of solvent and sonicated the samples again, without overnight incubation. Before extraction, we centrifuged the samples for 3 minutes at 3500 g. We recovered the supernatant and evaporated it under a nitrogen stream. We retained the dried pellets for bulk isotope analysis (lipid-extracted) and amino acid isotope analysis for a subset of individuals. We transesterified the extracted lipids by adding 3 mL of methanolic HCl (3 N) to each sample and incubating them at 60 °C for 15 minutes. We extracted fatty acid methyl esters (FAMEs) by adding 2 mL of hexane, mixing thoroughly, and transferring the upper organic phase to new vials. We repeated this step once per sample. We then pooled the hexane fractions, evaporated them to dryness under nitrogen, and re-dissolved the FAMEs in 700 µL of hexane.

We injected 1 µL of each sample into a gas chromatograph (GC-2010 Plus, Shimadzu) equipped with a Rtx-225 capillary column (50% cyanopropylmethyl–50% phenylmethyl polysiloxane; 30 m × 0.25 mm × 0.25 µm; Restek). We programmed the oven temperature as follows: hold at 60 °C for 1 minute; increase by 30 °C/min to 150 °C; increase by 3 °C/min to 170 °C; increase by 2 °C/min to 220 °C; hold at 220 °C for 6 minutes.

We identified FAMEs by comparing their retention times with those of a custom FAME+ reference mix. This mix included the Supelco 37-component FAME mix (ref: 18919-1AMP), supplemented with stearidonic acid methyl ester (C18:4n3; Supelco, ref: SMB00291) and mead acid ethyl ester (C20:3n9; Enzo Life Sciences, ref: BML-FM004). We identified a total of 37 fatty acids ranging from C6:0 to C24:1, with co-elution of C18:1n9 cis and trans, and C21:0 and C20:3n3c. We used the FAME+ mix to confirm retention times and assess response linearity across days and within analytical sequences. We also monitored the internal standard (C23:0 ME), which showed a typical standard deviation of ~15% across samples.

Supplemental Text 4: Bulk isotope analysis

We measured $\delta^{13}C$ and $\delta^{15}N$ values using a Vario PYRO CUBE elemental analyser (Elementar, Langenselbold, Germany) coupled to an IsoPrime 100 isotope ratio mass spectrometer (Elementar, Langenselbold, Germany). We weighed approximately 0.7 mg of lipid-extracted dorsal muscle tissue for each sample. We corrected stable isotope ratios for peak intensity drift across the analytical sequence when required. We normalized $\delta^{13}C$ and $\delta^{15}N$ values to international reference standards: Vienna Pee Dee Belemnite (VPDB) for carbon and atmospheric AIR for nitrogen. To ensure analytical accuracy, we used acetanilide reference materials with certified isotopic compositions (Schimmelmann, Indiana University). The standard deviation of the acetanilide standard remained below 0.10‰ for both $\delta^{13}C$ and $\delta^{15}N$ throughout all analytical runs. We verified the efficiency of lipid extraction by measuring carbon-to-nitrogen (C:N) mass ratios, which remained consistently close to proteinaceous values (mean: 3.1, sd: 0.0, range: 2.9-3.2), indicating minimal residual lipids in all samples. As a result, we did not apply corrections for lipid-related $\delta^{13}C$ depletion (*101*).

Supplemental Text 5: Analysis of amino acid stable isotopes

We weighed an amount of ~5 mg of the lipid-extracted biopsies, and hydrolyzed the samples with 2 mL 6M HCl at 110°C for 20 h. Because glutamine and asparagine are deaminated during the hydrolysis to glutamic acid and aspartic acid, here we report isotopic composition for Glx (glutamic acid + glutamine) and Asx (aspartic acid + asparagine). For fish from QL1 (N=9), the samples were not extracted for lipids before, as the data was part of another study. Importantly, lipid extraction does not affect the isotopic composition of organism tissue (*102*).

We derivatized the free AA to N-Acetyl Methyl Ester (NACME-AA) derivatives following previous protocols (*31*, *103*). Briefly, we first extracted the apolar compounds using 2 ml n-hexane/DCM. We added 0.1-1 mL (depending on the sample concentration) norLeucine 4.35 µMol as an internal standard. Prior to derivatization, the HCl of the hydrolyzed samples was evaporated at 110°C under N2. Subsequently, we methyl-esterified carboxylic groups using an excess of acidified methanol (6:1 anhydrous methanol:acetyl chloride) for 60 min at 75°C. Afterwards, amine groups were acetylated using an excess of acetylation mixture (1:2:5 acetic anhydride:triethylamine:acetone) for 10 min at 60°C. Before and after each reaction, we flushed the vials with N2 and dried excess solvent under N2 at 60°C, respectively. We dissolved the samples in 2mL ethyl acetate and removed precipitates and salt using 1 mL of saturated NaCl solution. We dried the ethyl acetate solution in which the NACME-AA were dissolved under N2 and brought them to a final volume of ~300 µL in GC vials.

We separated AAs on an Agilent 6890 Series Gas-Chromatography System equipped with a Gerstel MultiPurposeSampler MPS2 (Gerstel, Mühlheim, Germany) and an InertCap 35 GC column (GL Sciences, 60 m X 0.32 mm X 0.50 mm), using the following temperature program: 1 min at 80°C, 20°C/min until 135°C, 5°C/min until 160°C, hold for 3 min, 5°C/min until 280°C, hold for 5.25 min (total: 40 minutes).

We determined the carbon isotopic ratio of AAs using Delta V (or Delta V + Advantage) (Thermo) coupled to a GC and combustion tube. We ran every sample in duplicate ($\delta^{13}C$), or triplicate or more ($\delta^{15}N$). We ran AA in-house standards alongside the samples at the beginning, at the end, and after every 5 samples of each sequence. Before and after each AA standard, we ran an external caffeine standard of known isotopic composition (Schimmelmann, University of Indiana). To account for the isotopic kinetic effect of the carbon added during derivatization, we calculated correction factors using AA standard measurements following the formula in (*103*). We calculated standard deviation based on replicated measurements, and propagation of uncertainty due to carbon addition for $\delta^{13}C$ (*103*). We reported the values for 10 AAs and 9 AAs, for carbon and nitrogen, respectively. For each batch, we compared the in-house standard mean measurements to an in-house standard set as reference ($\delta^{13}C$), or known isotopic composition ($\delta^{15}N$). We checked that measured values were within the range of reference values, considering standard deviations. The standard deviation of the in-house amino acid standard was 0.7‰ for carbon (after accounting for carbon added during derivatization; N = 22, ranging from 0.4‰ for alanine to 1.3‰ for proline) and 0.8‰ for nitrogen (N = 35, ranging from 0.6‰ for valine to 1.1‰ for Asx).

Supplemental Text 6: Mixing model of marine subsidy reliance based on bulk isotopes

We implemented a mixing model of marine subsidy reliance via cannibalism on anadromous char eggs in MixSIAR (*81*), similar to (*60*).

We used bulk $\delta^{13}C$ and $\delta^{15}N$ as biomarkers because they strongly differentiate freshwater and marine resource use in our system, whereas fatty acids are more difficult to quantitatively integrate as biomarkers because they are regulated in consumers (*84*). (*60*) showed that a mixing model using bulk $\delta^{13}C$ alone was strongly aligned with estimates from essential amino acid mixing models, thereby reflecting the assimilation of marine resources through egg cannibalism. Here, we additionally included $\delta^{15}N$ because the $\delta^{15}N$ baseline is less variable in lake systems than in rivers, allowing us to add a second dimension that strongly differentiated lake resources from marine ones.

We used resident char and anadromous char as integrated baselines for freshwater and marine subsidy reliance, respectively. Resident char were defined as individuals from lakes without migration. By doing so, we encompassed the isotopic variability integrated by char without marine influence. Because the $\delta^{13}C$ baselines differed strongly among lake stages (see Results), we ran three separate mixing models for the three stages (i.e, glacial, recent and old). We integrated the sources as end-members by subtracting trophic fractionation values of $\delta^{13}C$ (0.5‰) and $\delta^{15}N$ (3.3‰) from the consumer values (*104*). Including anadromous char as marine subsidy end-members is appropriate because char directly feed on their eggs, which have a similar isotopic composition (*60*). We included anadromous char from all lakes, as their bulk isotopic composition did not differ strongly among lakes, but filtered for adult individuals (with semi-developed or developed gonads) to exclude young migrants with distinct isotopic signatures. We included both males and females to increase source sample size and capture the natural variation in anadromous feeding populations.

We considered trophic fractionation between char and their prey of 0.50 ± 0.13‰ for $\delta^{13}C$ and 3.3 ± 0.26‰ for $\delta^{15}N$ (mean ± SD; (*104*), which are common values for high-level consumers). We included all char in the model to use residents without fjord access as a negative control. We included char individual ID as a random factor to capture individual variation in resource use (*105*). Char ID was nested within connection to the fjord (yes/no), which was included as a fixed factor, as the presence of migration would necessarily influence marine subsidy reliance.

We included both residual and process errors (*106*). We used the "normal" run setting in MixSIAR with three chains of 400,000 iterations, a burn-in of 300,000 iterations, and a thinning interval of 100, resulting in 1,000 posterior samples per chain. We assessed model convergence using Gelman, Heidel, and Geweke diagnostics.

Supplemental Text 7: Mixing model of primary producers based on essential amino acid $\delta^{13}C$

We used the MixSIAR package, which is the recommended approach for making quantitative estimates from $\delta^{13}C$ essential amino acid (EAA) fingerprinting (*82*, *107*).

We used five commonly reported EAAs: isoleucine (Ile), leucine (Leu), threonine (Thr), phenylalanine (Phe), and valine (Val), because they are unequivocally considered EAAs (*107*), and are the most commonly reported EAAs in the literature, allowing the use of published reference values. We mean-normalized their $\delta^{13}C$ values to represent the fingerprinting signal.

We used literature data from the main primary producer phyla that could contribute nutrients to the system: photosynthetic organisms (including algae and cyanobacteria, hereafter "algae"), non-photosynthetic bacteria (hereafter "bacteria"), and vascular plants (hereafter "plants"). We focused on pure sources of primary production, rather than composite materials such as soil, to accurately quantify the relative contributions of each primary producer group (*107*). The algal dataset (N = 50) included a wide range of taxa from both cultures and field collections (*31*, *83*). Cyanobacteria (N = 6) were included in the algal group because they are photosynthetic and exhibit similar fingerprinting patterns (*31*). The bacterial dataset (N = 34) consisted of culture-derived data (*31*, *83*, *108*). The plant dataset (N = 17) comprised wild samples from Alaska and Denmark (*31*), as well as cultured material (*108*).

We set discrimination factors to 0‰ with a standard deviation of 0.5‰, reflecting minimal fractionation and analytical uncertainty. Individual char were included as a random effect (*105*), and uninformative priors were used. We included both residual and process errors (*106*). We used the "normal" run setting in MixSIAR with three chains of 400,000 iterations, a burn-in of 300,000 iterations, and a thinning interval of 100, resulting in 1,000 posterior samples per chain. We assessed model convergence using Gelman, Heidel, and Geweke diagnostics.

Differences in fingerprinting among char were visualized using principal component analysis (PCA).

Supplemental Text 8: Trophic position estimates from CSIA-AA

We calculated TP using the relative ^{15}N-enrichment of Glx over Phe, calculating TP as follows (*86*):

$$TP_{Glx\text{-}Phe} = (\Delta^{15}N_{Glx\text{-}Phe} - \beta_{Glx\text{-}Phe}) / TDF_{Glx\text{-}Phe} + 1$$

where $TDF_{Glx\text{-}Phe}$ and $\beta_{Glx\text{-}Phe}$ represent the relative trophic enrichment of Glx over Phe in consumers and the isotopic offset in primary producers, respectively. We used a $TDF_{Glx\text{-}Phe}$ of +5.7 ‰ (sd=0.3) and $\beta_{Glx\text{-}Phe}$ of +3.6 ‰ (sd=0.5) , which are mean values inferred empirically from marine fish that showed high predictability with fish stomach content (*87*), and are considered more realistic for estimating fish TPs in natural food webs. The β value is not different from the commonly reported value for aquatic primaray producers, including algae and bacteria (*109*). We included propagation of uncertainties of $TDF_{Glx\text{-}Phe}$,$\beta_{Glx\text{-}Phe}$, and $\Delta^{15}N_{Glx\text{-}Phe}$ analytical precision (estimated for each individual with three replicates) using 1000 Monte

Carlo simulations in the *propagate* package (*110*). We reported mean TP, TP standard deviation, and 5–95% range.

FIGURES

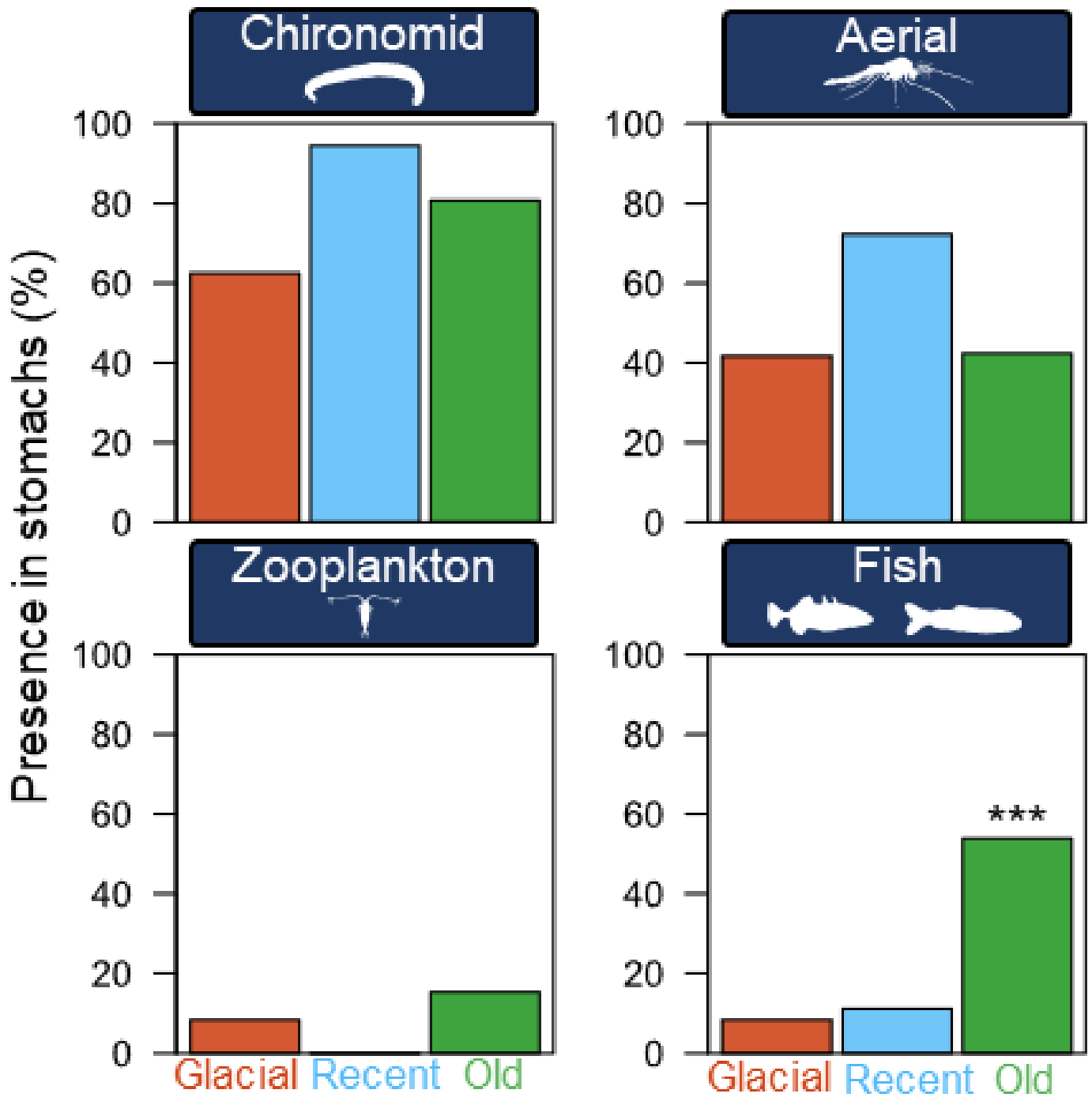

Figure S1. Char stomach contents. Presence in char stomachs (as % of total) for glacial (n = 24), recent (n = 18), and old lakes (n = 26). Stomach contents were categorized into four groups: chironomids (chironomid larvae), aerial (all invertebrates in the aerial phase), zooplankton, and fish (stickleback, char, char eggs, and unidentified fish). Stars indicate significance (***: $P < 0.001$) based on chi-squared tests with Holm adjustment for multiple comparisons.

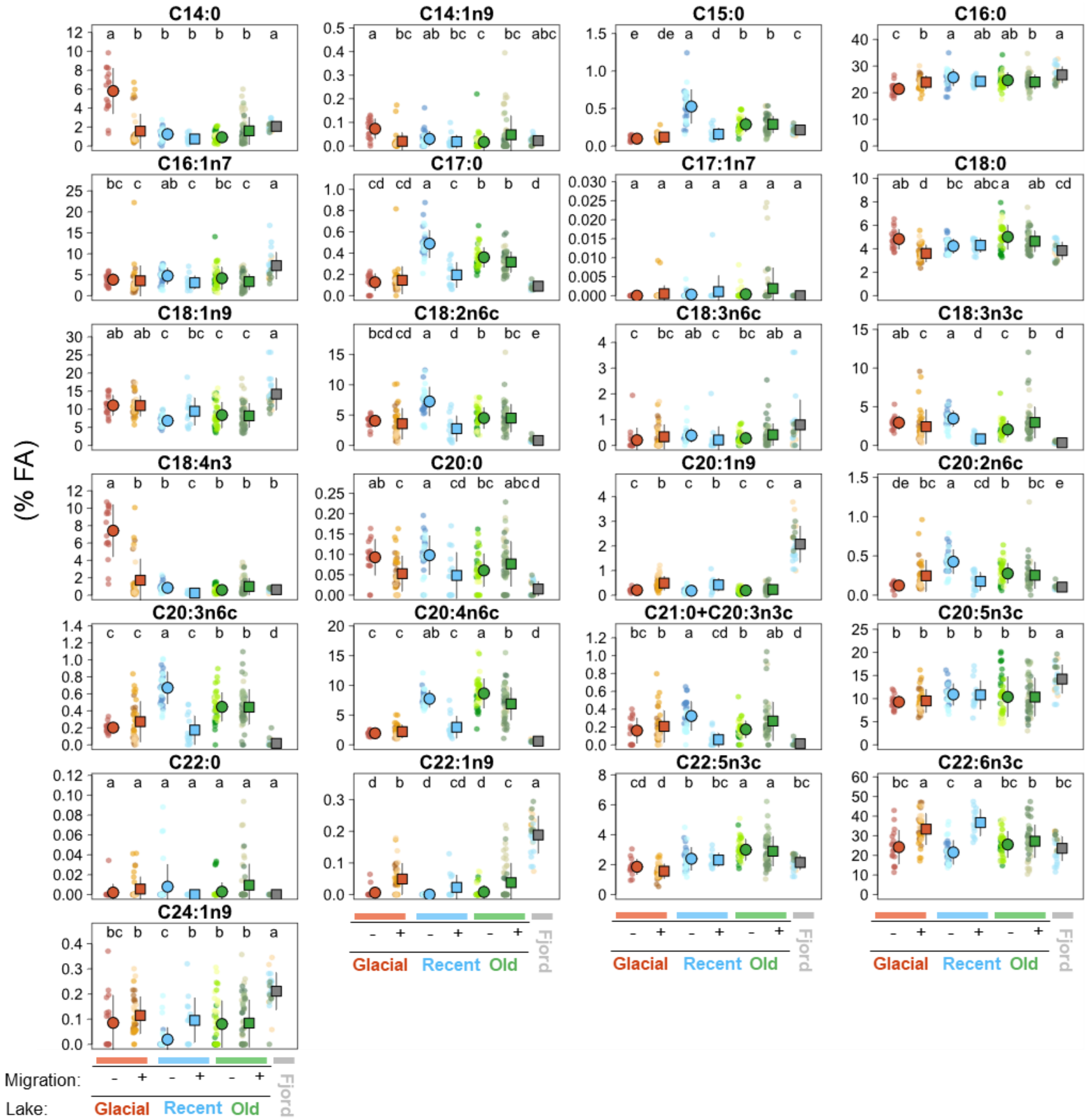

Figure S2. Fatty acid profiles of char across lake stages and fjord connectivity. Comparison of fatty acids (% FA) across lake stage (color), access to the fjord (no: circles; yes: squares), and fjord-feeding char (gray squares). Large dots show mean, and associated error bars show one standard deviation. Small dots represent individual char, with colors indicating lake affiliation (Main Text, Figure 1). Different letters denote significant differences among groups (pairwise Kruskal–Wallis tests with Holm correction).

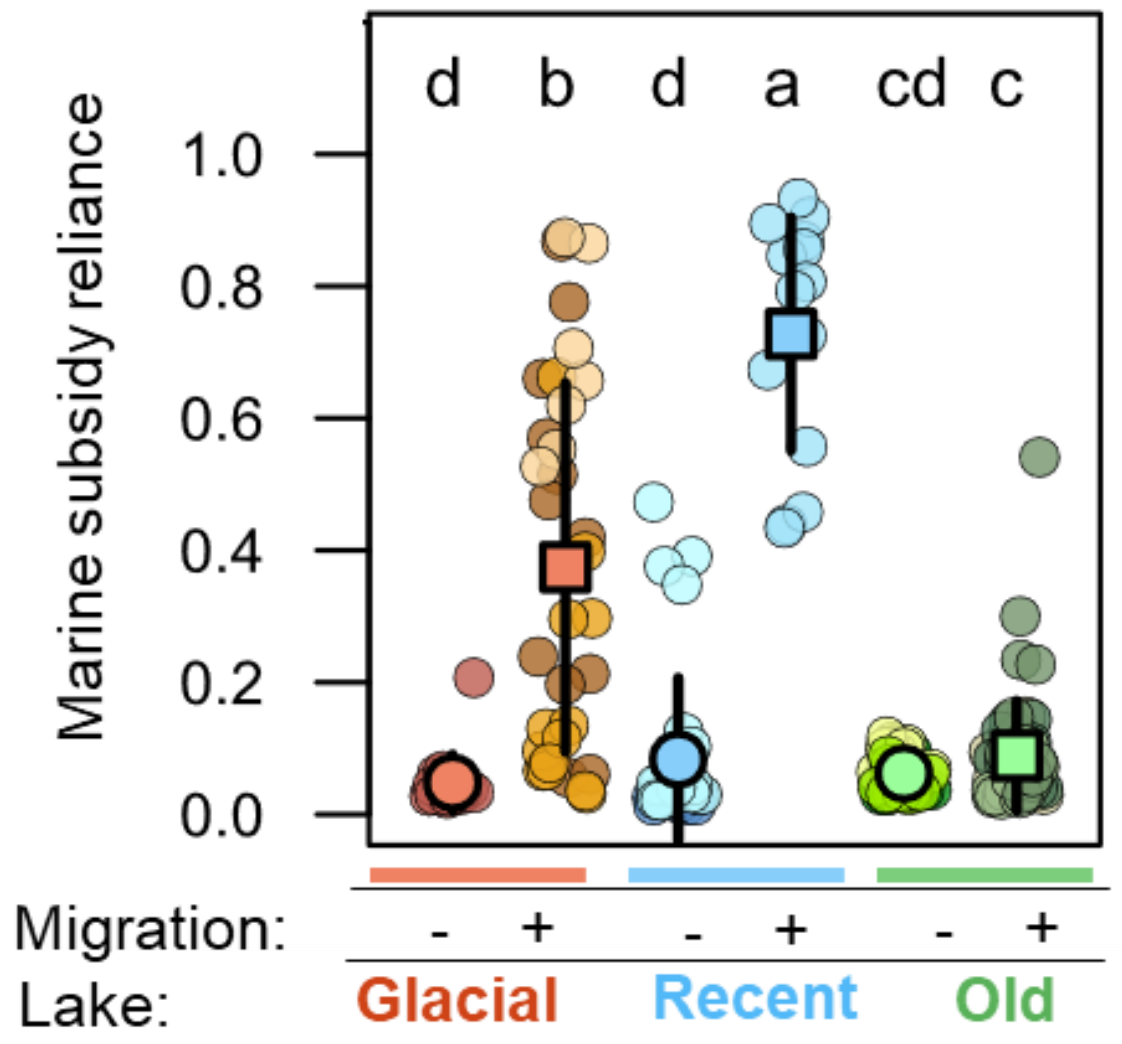

Figure S3. Marine subsidy reliance via egg cannibalism. Comparison of marine subsidy reliance by egg cannibalism across lake stage (color), access to the fjord (no: circles; yes: squares). Large dots show mean, and associated error bars show one standard deviation. Small dots represent individual char, with colors indicating lake affiliation (Main Text, Figure 1). Different letters denote significant differences among groups (pairwise Kruskal–Wallis tests with Holm correction).

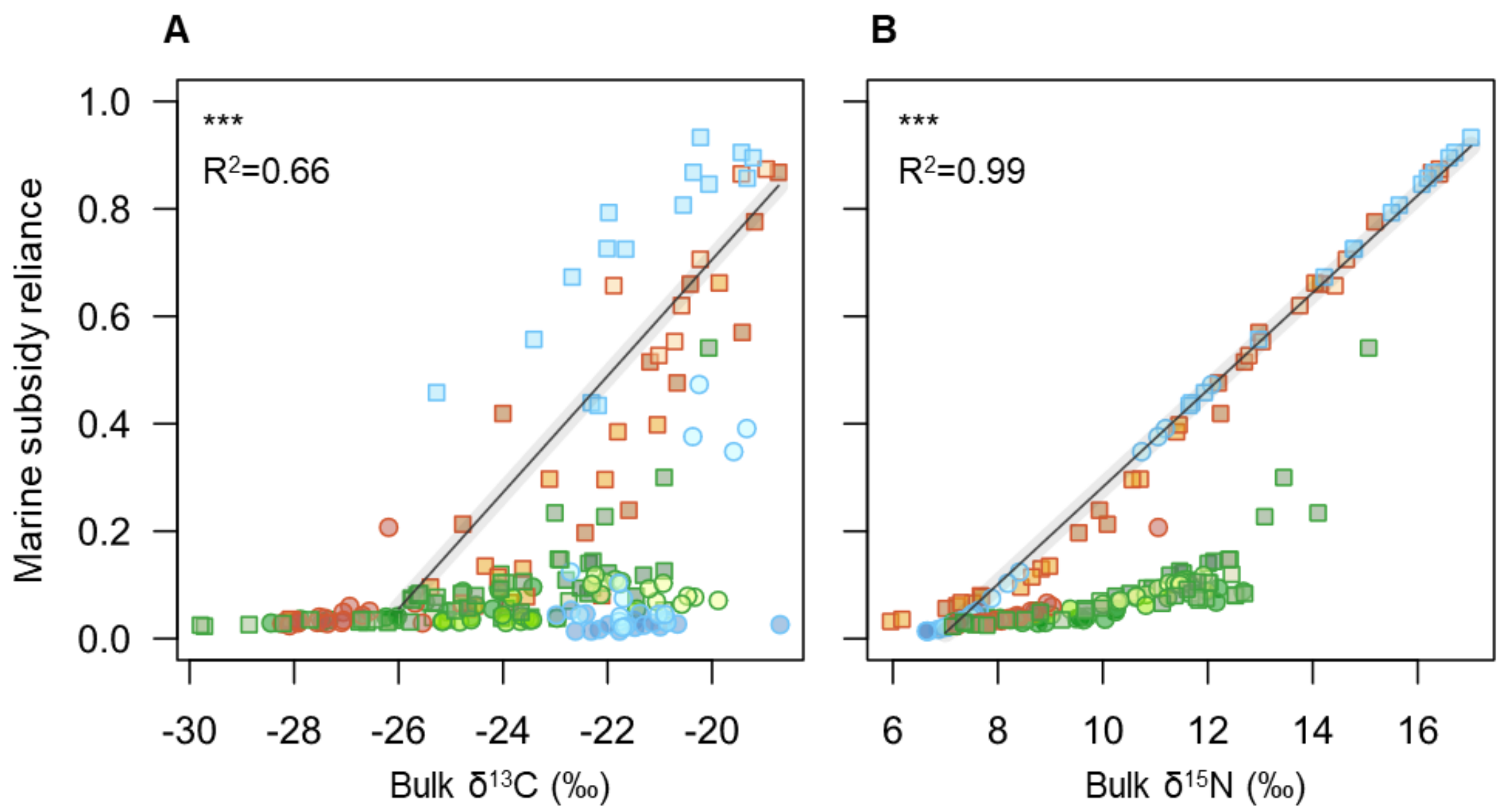

Figure S4. Relationship between marine subsidy reliance and bulk isotopes. Relationship between marine subsidy reliance by egg cannibalism (y-axis) and (A) bulk $\delta^{13}C$, and (B) bulk $\delta^{15}N$. Each point represents an individual char, with colors indicating lake affiliation (see Main Text, Figure 1) and shapes indicating fjord access (no: circles; yes: squares). Lines show the linear relationship for char in glacial and recent lakes with fjord access, along with the associated R^2 and p-values. Some individuals without fjord access (circles) show non-zero marine subsidy reliance, reflecting false negatives, particularly in recent lakes where the $\delta^{13}C$ baseline is more similar to marine baselines.

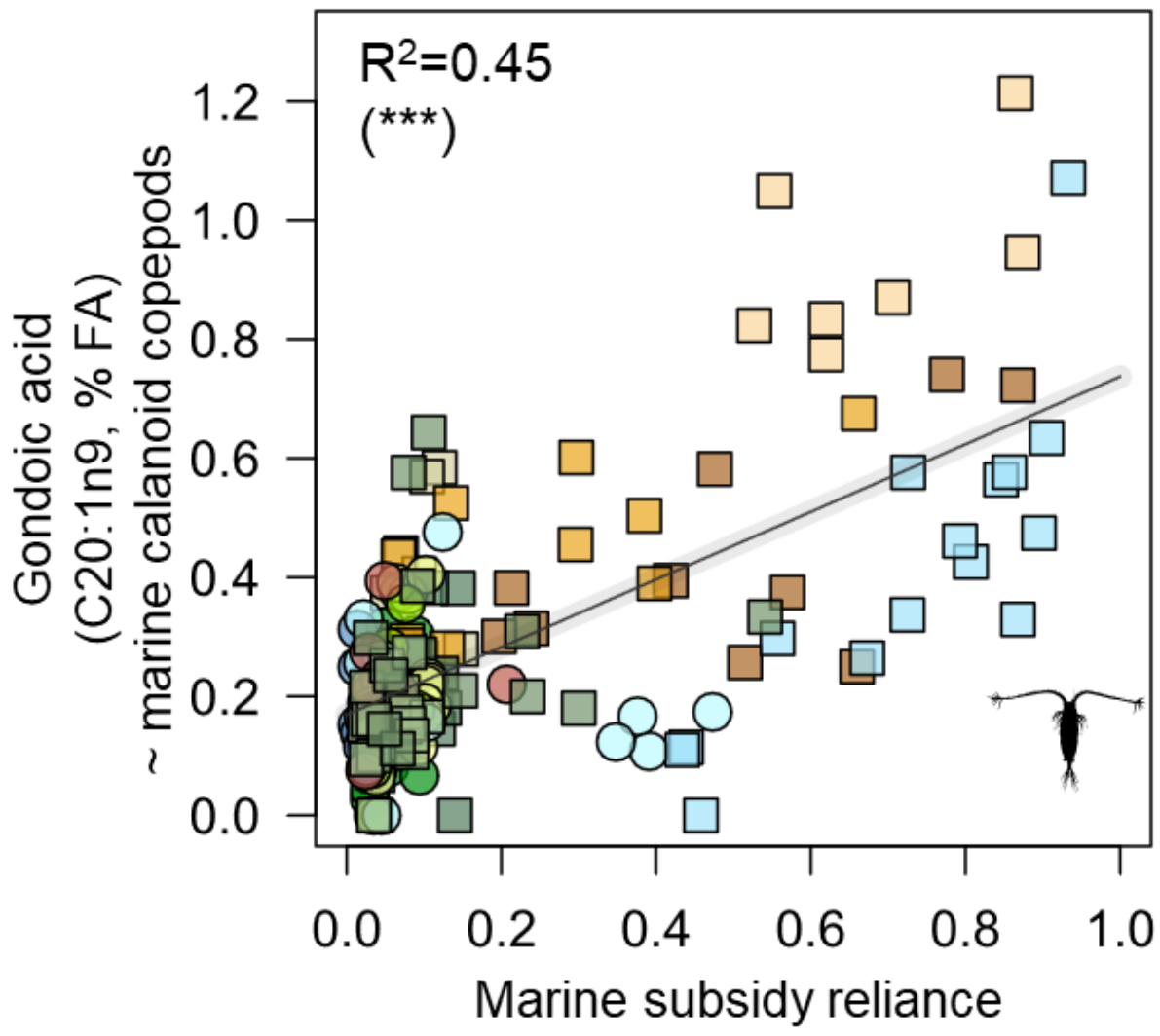

Figure S5. Relationship between marine subsidy reliance and C20:1n9. Relationship between marine subsidy reliance by egg cannibalism, as inferred from a bulk isotope mixing model, and the relative abundance of C20:1n-9, a typical marine calanoid biomarker in the arctic (*75*, *95*). Dots represent individual char, with colors indicating lake and shapes indicating fjord access (circle = no, square = yes). The line shows the linear regression, with the associated P-value and R^2. Lower values of C20:1n-9 in char from recent lakes, despite a similarly high reliance on marine subsidies, might be linked to the lower abundance of C20:1n-9 in anadromous char from this lake (Fig. S2).

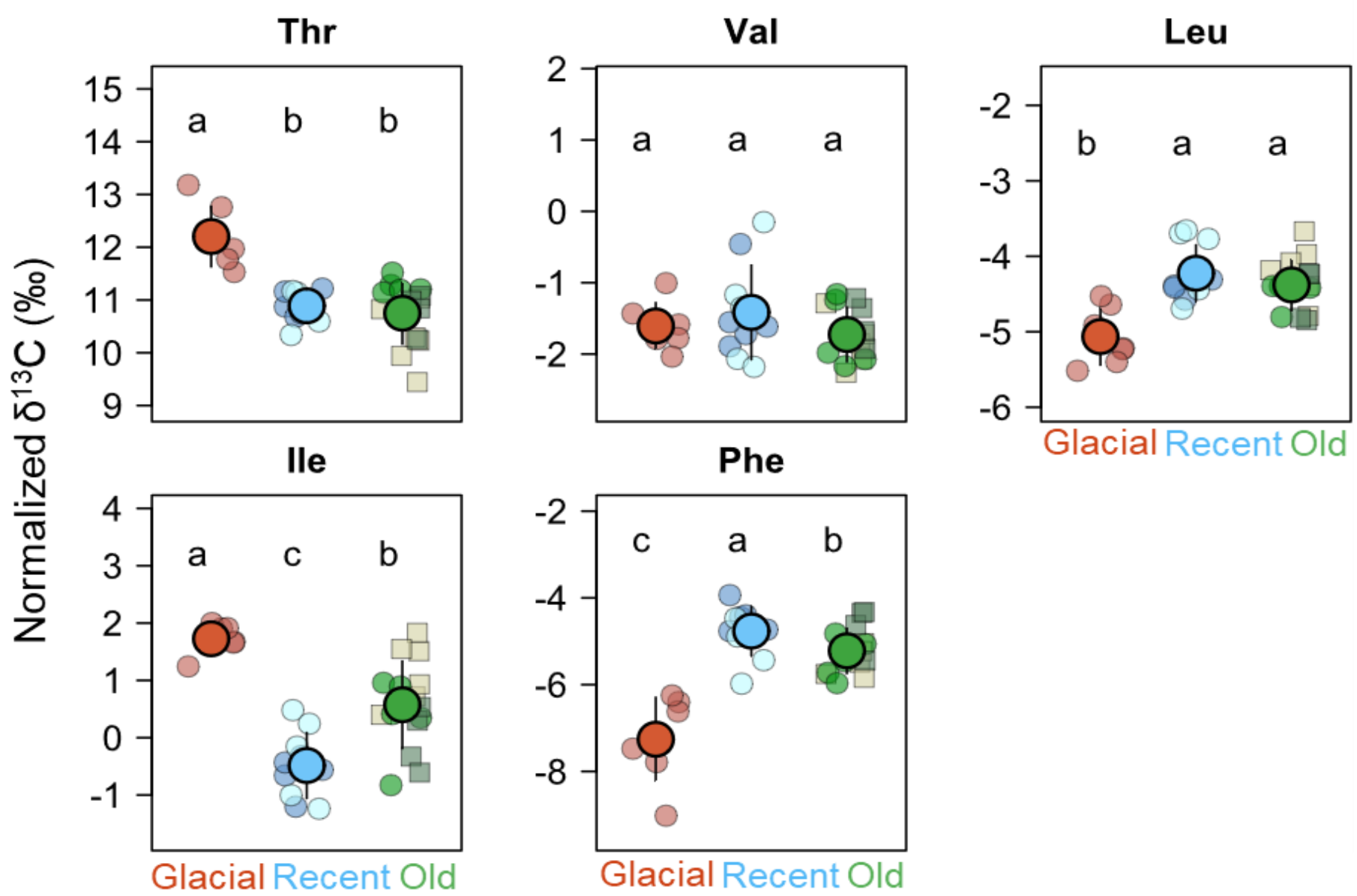

Figure S6. Normalized $\delta^{13}C$ values of essential amino acids. Normalized $\delta^{13}C$ values for the five essential amino acids (EAAs) used in the mixing model: threonine (Thr), valine (Val), leucine (Leu), isoleucine (Ile), and phenylalanine (Phe). Small dots represent individual char (color indicates lake, Figure 1; shape indicates fjord access: square = yes, circle = no). Large dots show stage means, with error bars representing one standard deviation. Different letters denote significant differences among lake stages (pairwise Kruskal–Wallis tests with Holm correction). This plot excludes systems with marine subsidy reliance by egg cannibalism (glacial and recent clear-water lake stages with fjord access, and one old lake, Ilua Tasia).

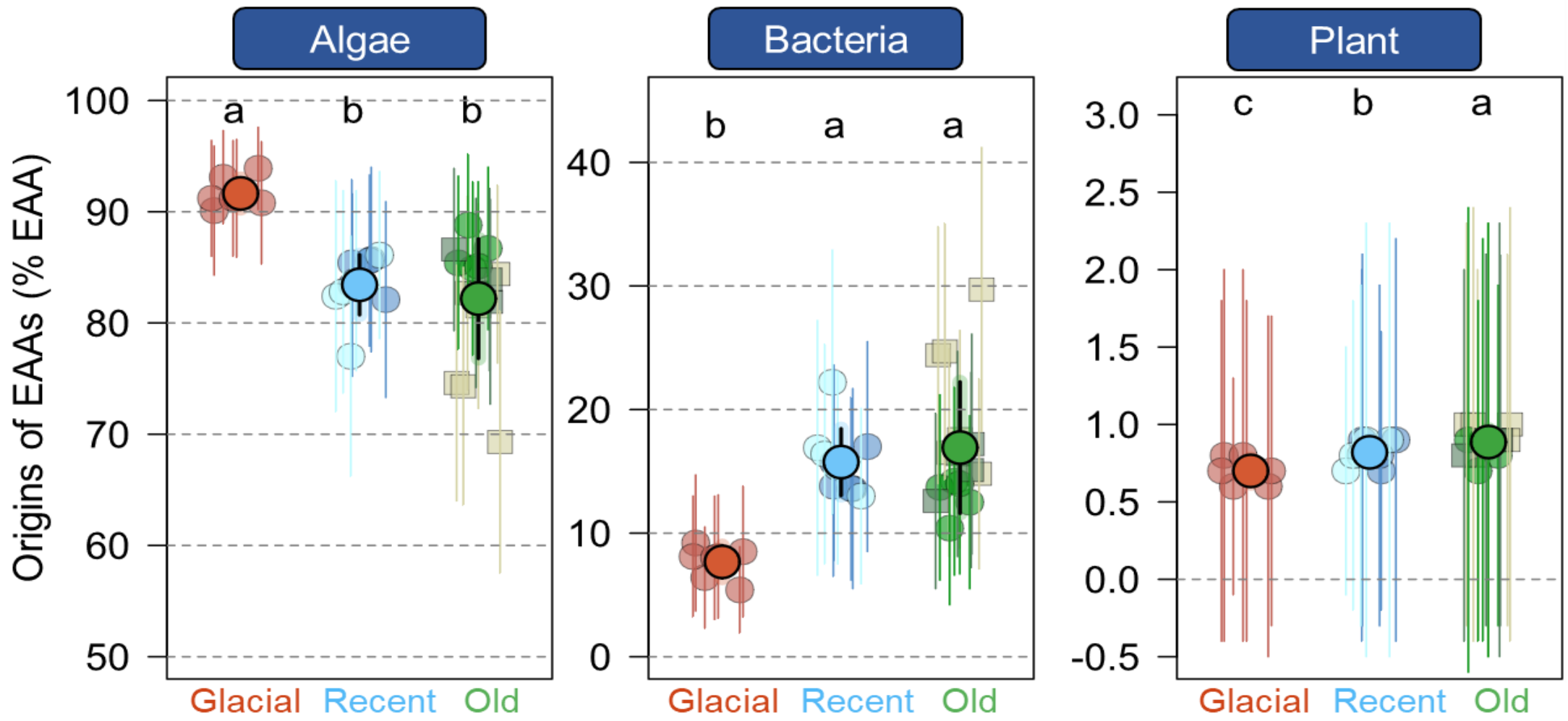

Figure S7. Primary producer reliance. Results of the Bayesian mixing model using δ^{13}C fingerprinting of five essential amino acids (threonine, valine, leucine, isoleucine, and phenylalanine; see Methods) and literature values, predicting EAA origins from algae (algae and cyanobacteria), bacteria (non-photosynthetic bacteria), and plants (vascular plants). Dots with light contours represent individual char, with error bars showing one standard deviation. Dots with dark contours represent stage means, with error bars showing one standard deviation. Different letters indicate significant differences among lake stages (pairwise Kruskal–Wallis tests with Holm correction). This plot excludes systems with marine subsidy reliance by egg cannibalism (glacial and recent clear-water lake stages with fjord access, and one old lake, Ilua Tasia).

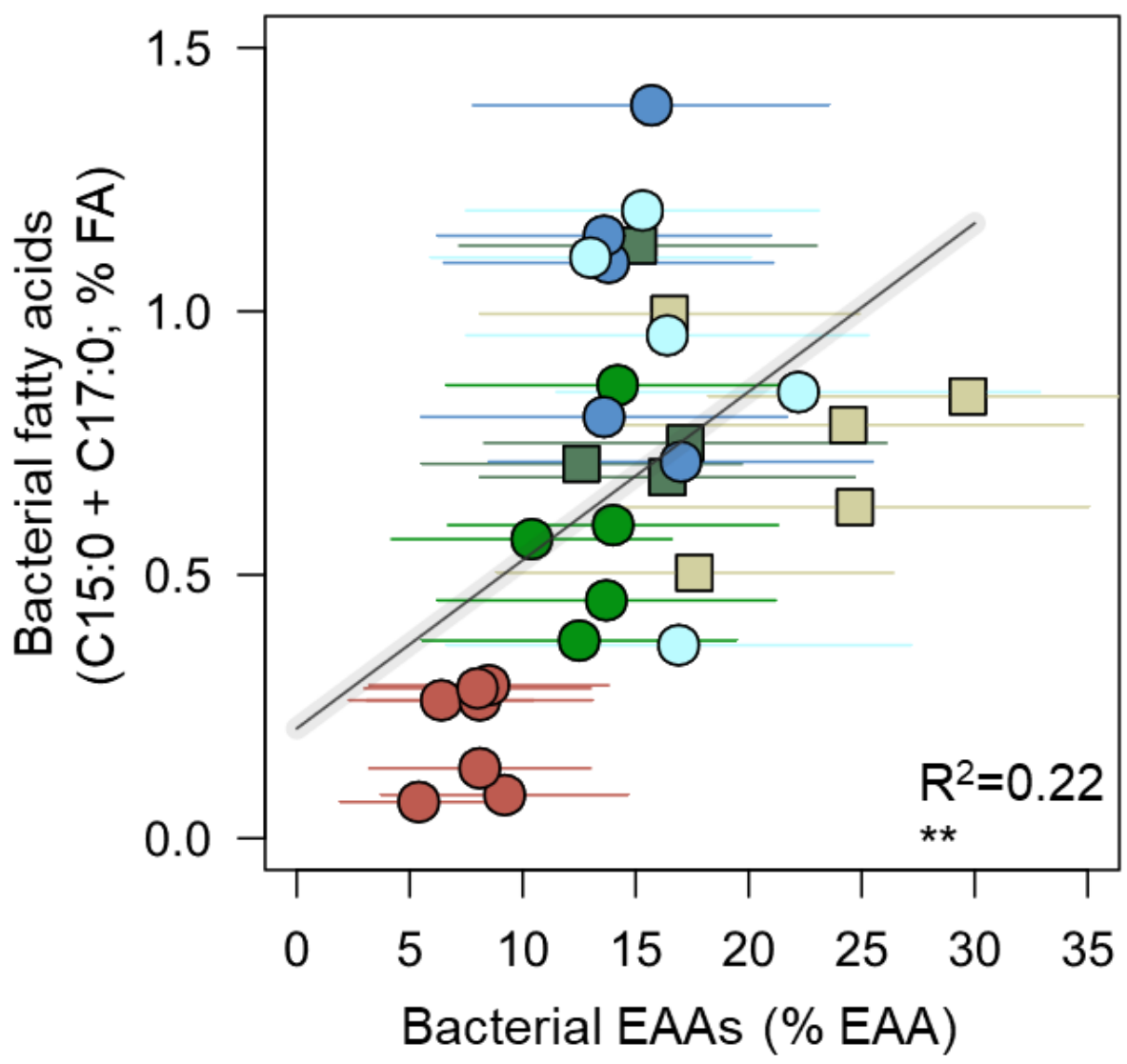

Figure S8. Relationship between bacterial EAA reliance and fatty acid bacterial markers. Relationship between bacterial fatty acids (C15:0 + C17:0; % FA) and bacterial contribution to essential amino acids (EAA δ^{13}C fingerprinting mixing model; see Methods). Dots represent individual char, with colors indicating lake and shapes indicating fjord access (circle = no, square = yes). Error bars show one standard deviation of the mixing model results. This plot excludes systems with marine subsidy reliance by egg cannibalism (glacial and recent clear-water lake stages with fjord access, and one old lake, Ilua Tasia).

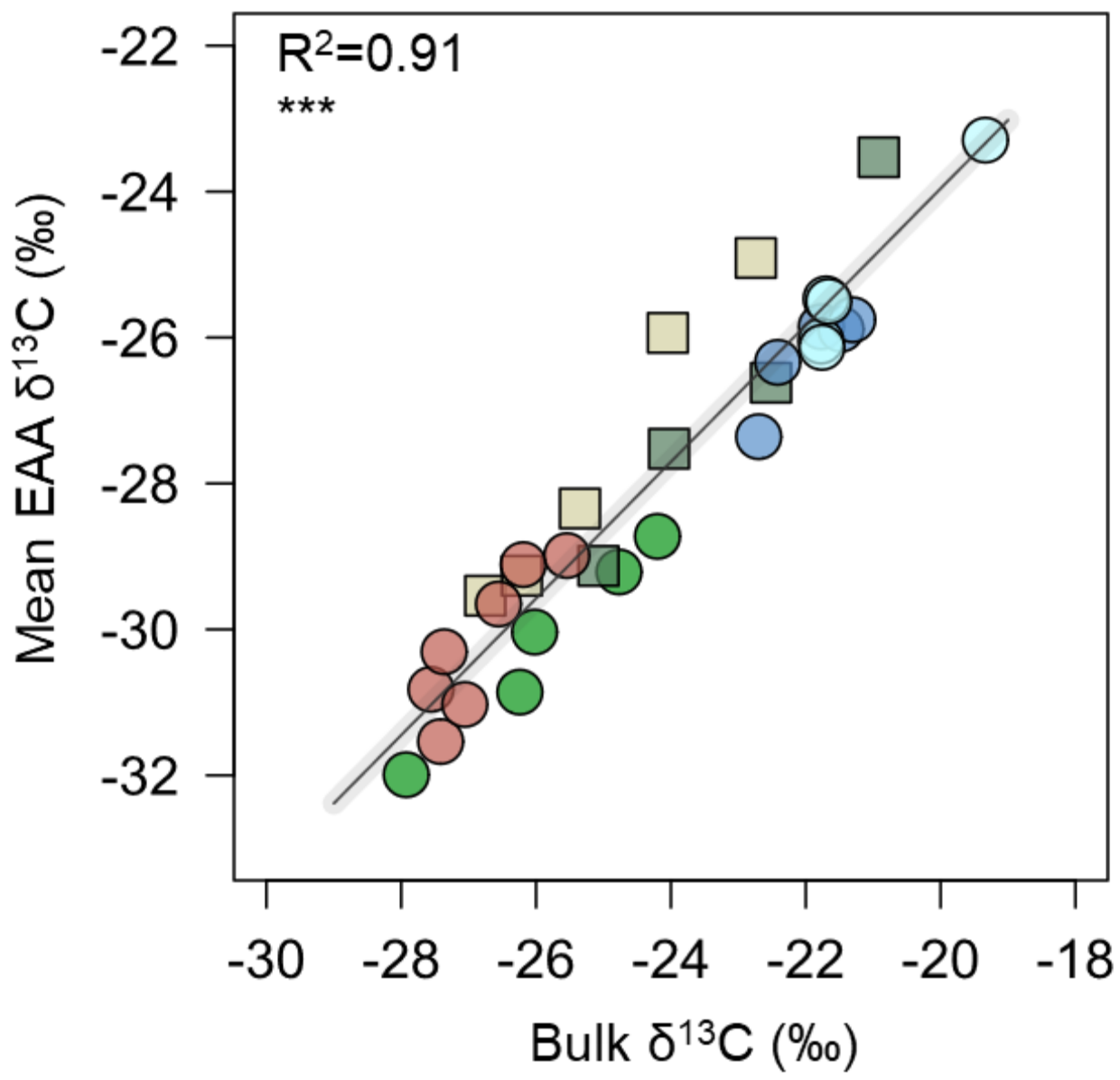

Figure S9. Relationship between bulk δ^{13}C and mean EAA δ^{13}C. Relationship between mean δ^{13}C of five essential amino acids (threonine, valine, leucine, isoleucine, and phenylalanine) and bulk δ^{13}C. Dots represent individual char, with colors indicating lake and shapes indicating fjord access (circle = no, square = yes). This plot excludes systems with marine subsidy reliance by egg cannibalism (glacial and recent clear-water lake stages with fjord access, and one old lake, Ilua Tasia).

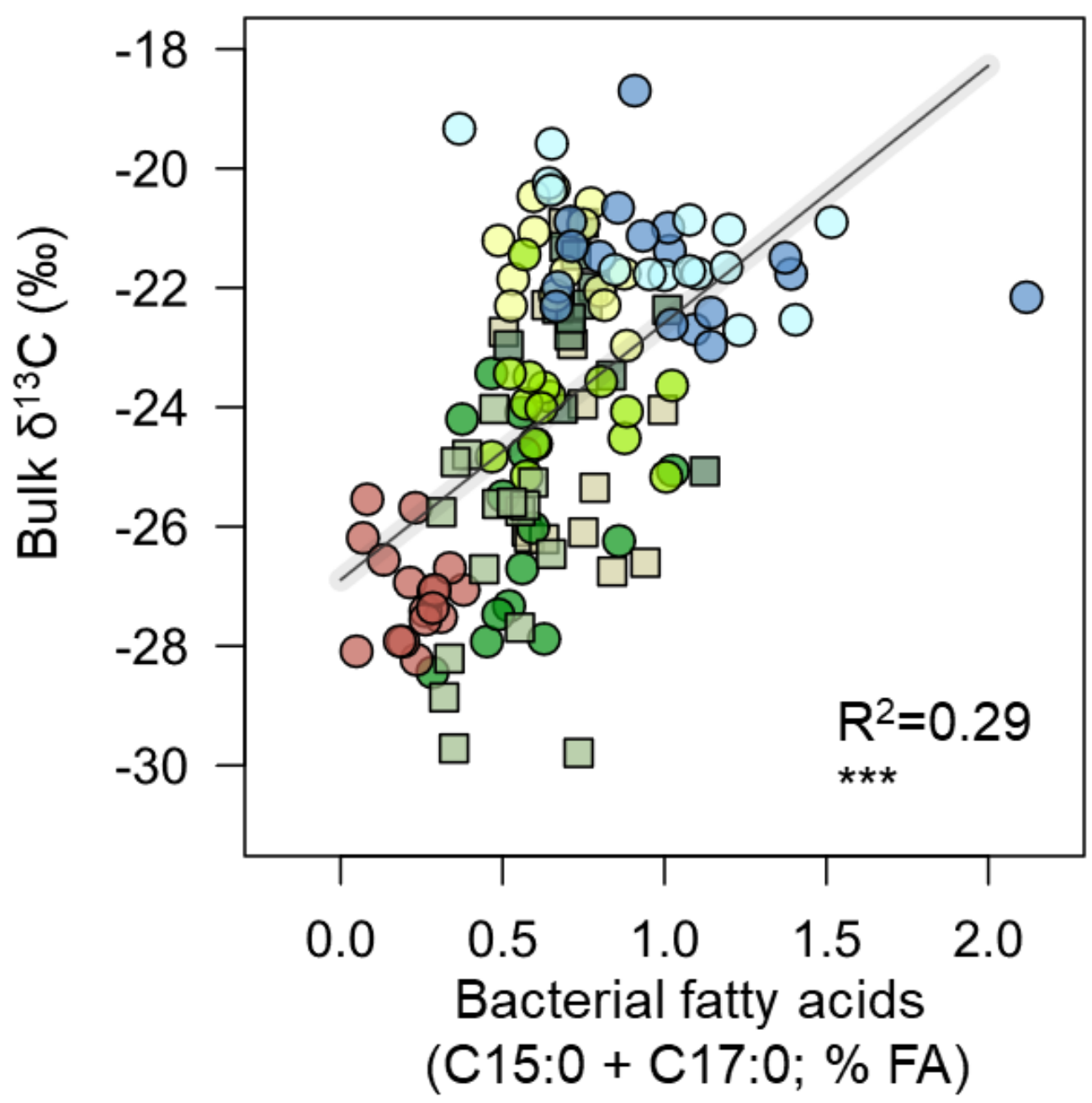

Figure S10. Relationship between bulk δ^{13}C and bacterial fatty acids. Relationship between bulk δ^{13}C and bacterial fatty acids (C15:0 + C17:0; % FA). Dots represent individual char, with colors indicating lake and shapes indicating fjord access (circle = no, square = yes). This plot excludes systems with marine subsidy reliance by egg cannibalism (glacial and recent clear-water lake stages with fjord access, and one old lake, Ilua Tasia).

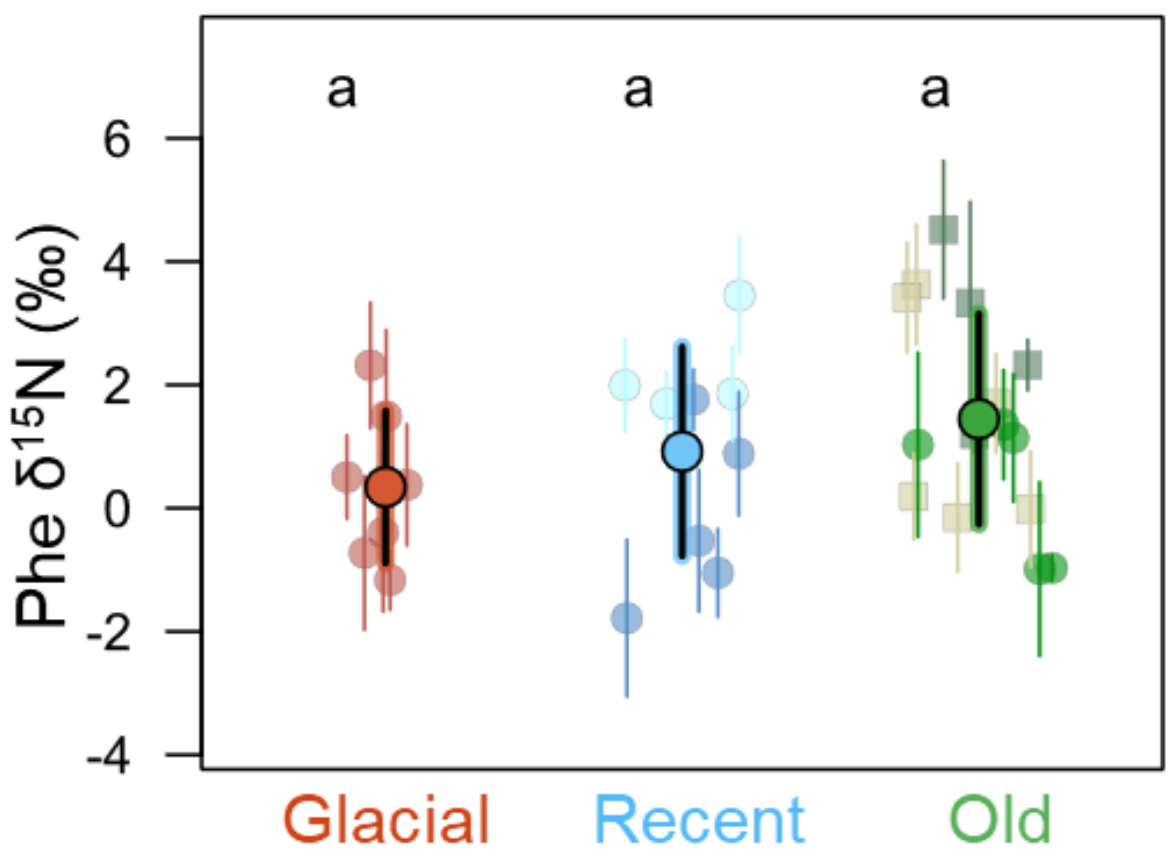

Figure S11. Baseline δ¹⁵N from source amino acid phenylalanine. Baseline δ¹⁵N, as inferred from source phenylalanine δ¹⁵N, across lake stages (see color). Dots with light contours represent individual char, with error bars showing one standard deviation (analytical precision). Dots with dark contours represent stage means, with error bars showing one standard deviation. Different letters indicate significant differences among lake stages (pairwise Kruskal–Wallis tests with Holm correction). This plot excludes systems with marine subsidy reliance by egg cannibalism (glacial and recent clear-water lake stages with fjord access, and one old lake, Ilua Tasia).

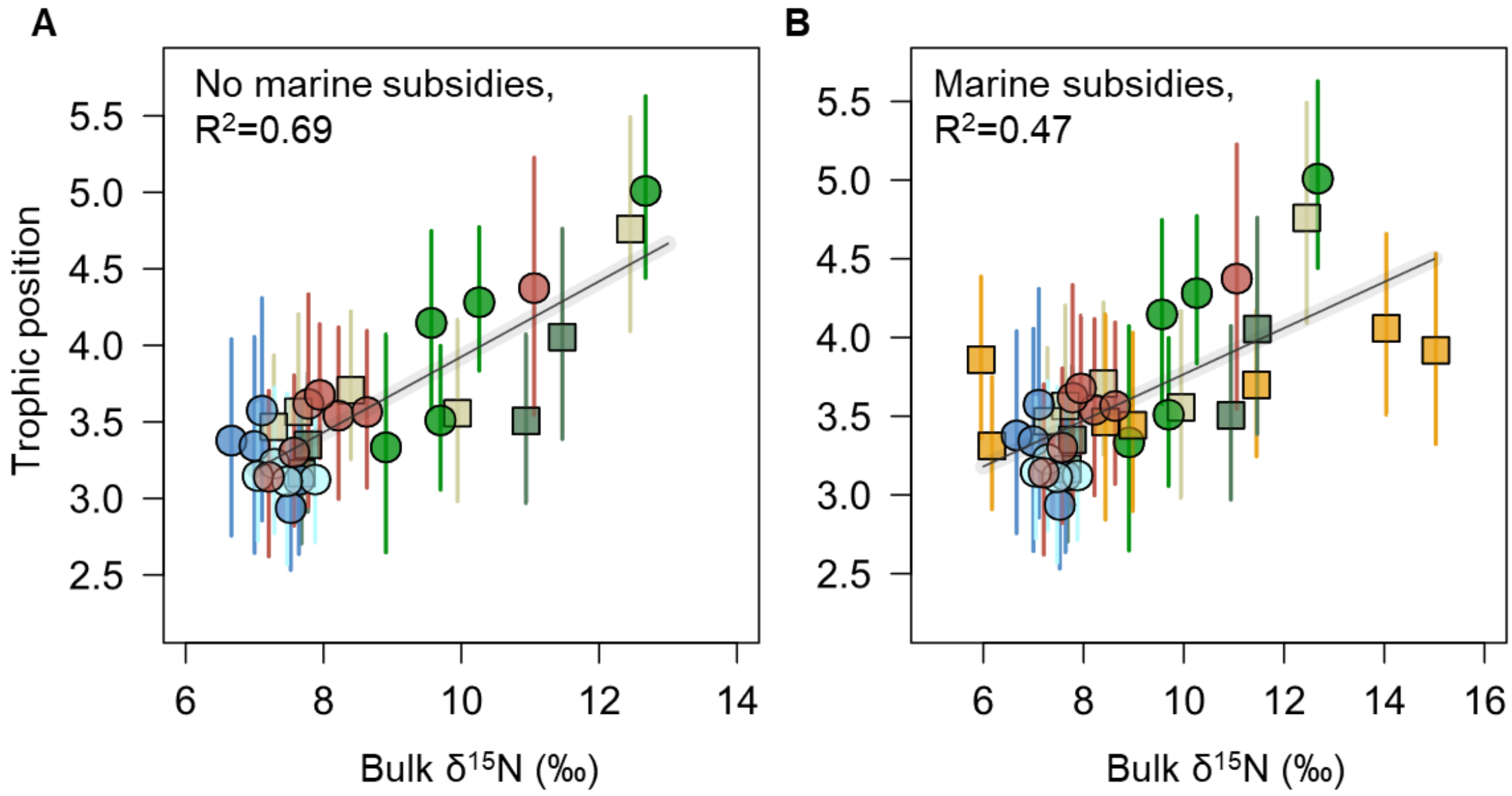

Figure S12. Relationship between CSIA-AA-based char trophic position and bulk δ¹⁵N. Relationship between char trophic position (inferred from the amino acid δ¹⁵N method; see Methods) and bulk δ¹⁵N. Dots represent individual char, with colors indicating lake and shapes indicating fjord access (circle = no, square = yes). Error bars show the 95% distribution of fish trophic position from Bayesian simulations. (A) This plot excludes systems with marine subsidy reliance by egg cannibalism (glacial and recent clear-water lake stages with fjord access, and one old lake, Ilua Tasia). (B) Shows the same relationship including all samples.

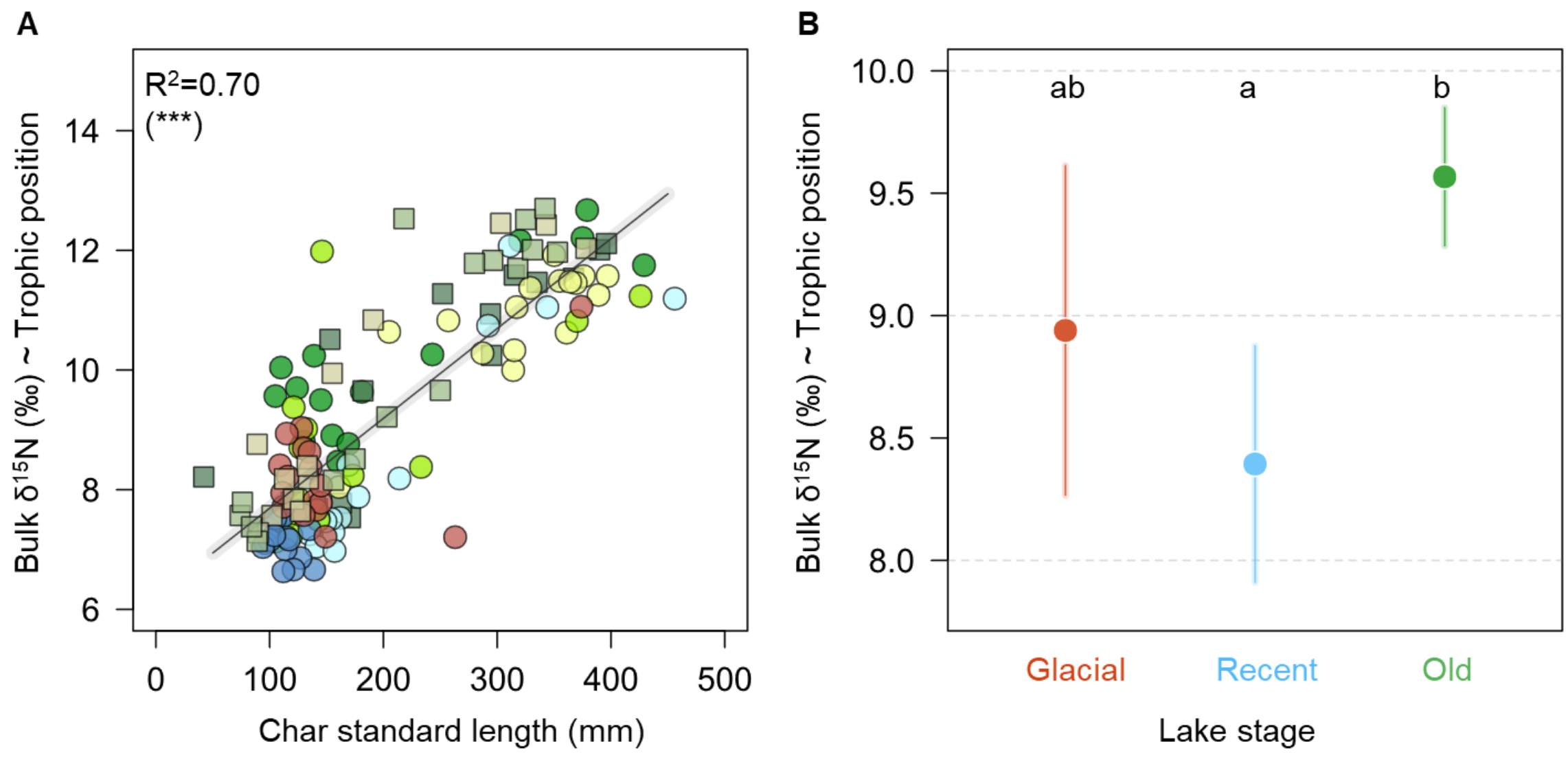

Figure S13. Changes in trophic position with char body size and lake stage. (A) Relationship between trophic position, as inferred from bulk δ¹⁵N, and char standard length for all char without marine subsidy reliance by egg cannibalism. Dots represent individual char, with shape indicating fjord access (yes: square; no: circle) and color indicating lake (see Main Text, Figure 1). The line shows the fitted relationship, with associated R^2 and significance. (B) Effect of lake stage on bulk δ¹⁵N values in char, estimated from a linear mixed-effects model including lake stage and char length as fixed effects and lake as a random factor. The plot shows size-corrected δ¹⁵N values across lake stages. Different letters indicate statistically significant differences among lake stages. This plot excludes systems with marine subsidy reliance (glacial and recent clear-water lake stages with fjord access, and one old lake, Ilua Tasia).

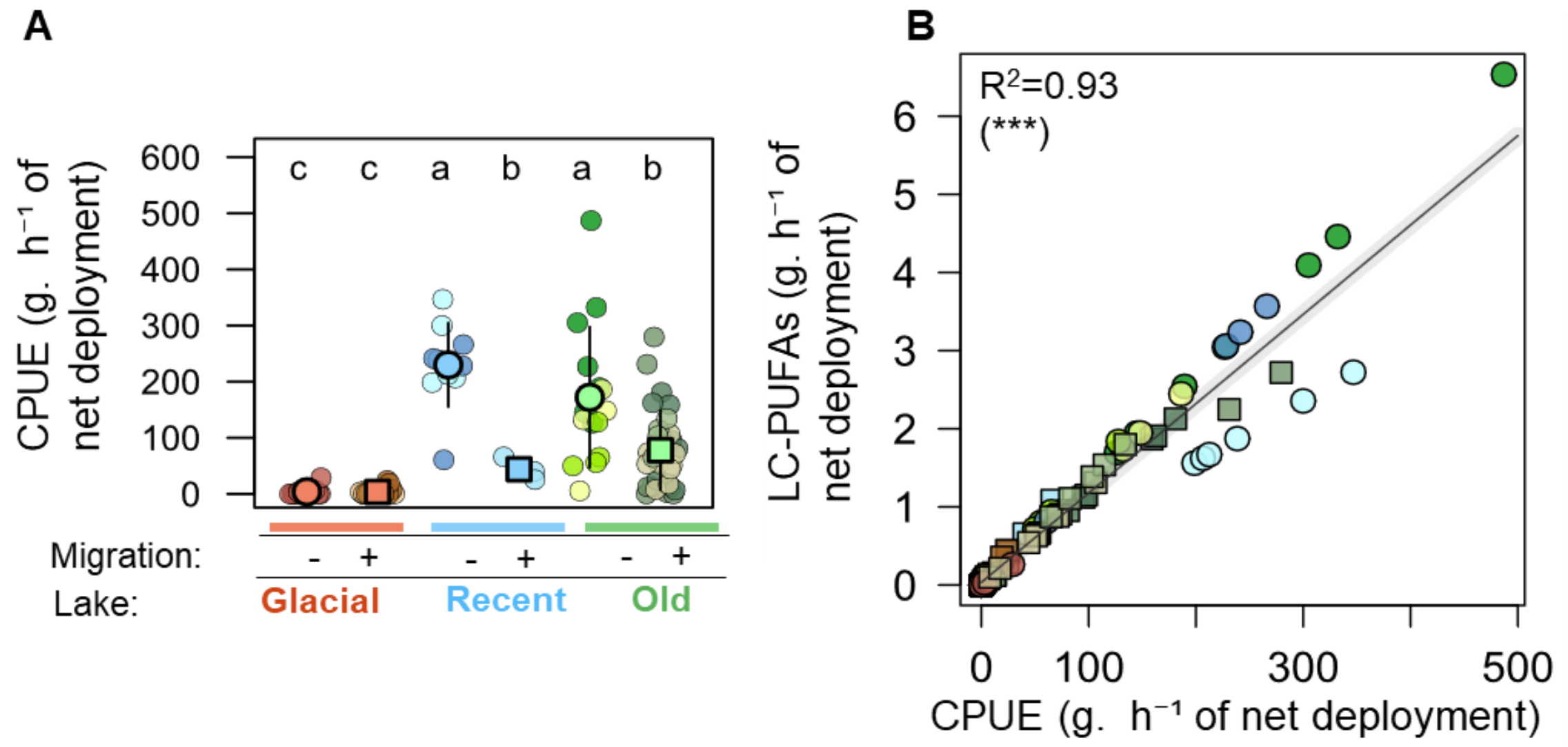

Figure S14. Char production (CPUE) and its relationship with total LC-PUFA production. (A) Total production of char across lake stages, as inferred from catch per unit effort (CPUE) (gram fish h^{-1} deployment). Symbols indicate fjord connectivity. Different letters indicate significant differences (pairwise Kruskal–Wallis test, $P<0.05$). (B) Relationship between total LC-PUFA production and total fish biomass production. The line shows the fitted relationship, with associated R^2 and significance.

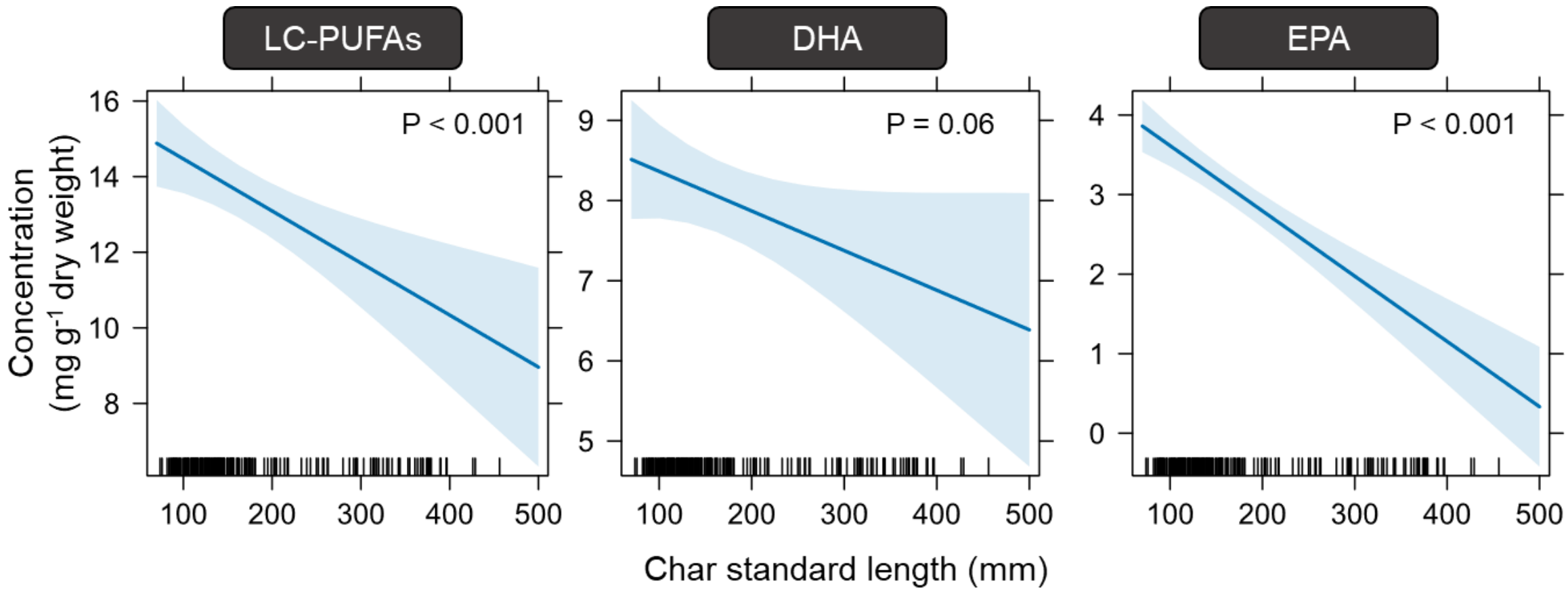

Figure S15. Effect of char body size on the concentrations of LC-PUFAs, DHA, and EPA. Partial residual plots of the glm models showing the effect of char standard length on the concentration in char of LC-PUFAs, DHA, and EPA, and their associated P-values. The line shows the mean, and the blue shaded area represents the 95% confidence interval. Rug lines indicate the size distribution of char.

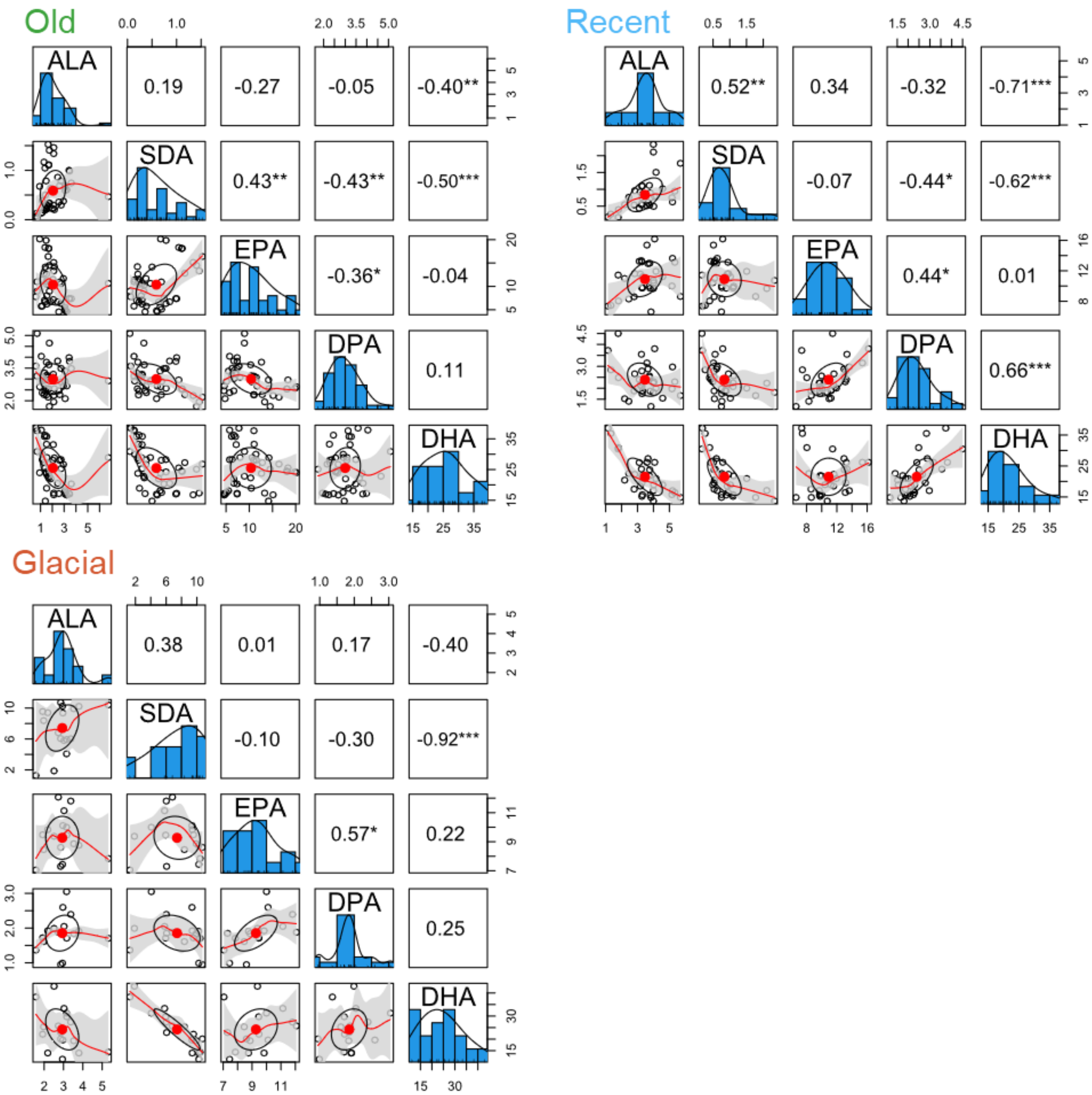

Figure S16. Correlation matrix between DHA relative abundance and its precursors across lake stages. Correlations between the relative abundance (% FA) of docosahexaenoic acid (DHA, 22:6n-3) and its precursors: alpha-linolenic acid (ALA, 18:3n-3), stearidonic acid (SDA, 18:4n-3), eicosapentaenoic acid (EPA, 20:5n-3), and docosapentaenoic acid (DPA, 22:5n-3), in individual char. Panels show results from lakes grouped by glacial influence, all without fjord connectivity to avoid confounding effects of marine-derived fatty acids. The upper panels display Pearson correlation coefficients, with significance indicated by asterisks: ***p < 0.001, **p < 0.01, *p < 0.05.

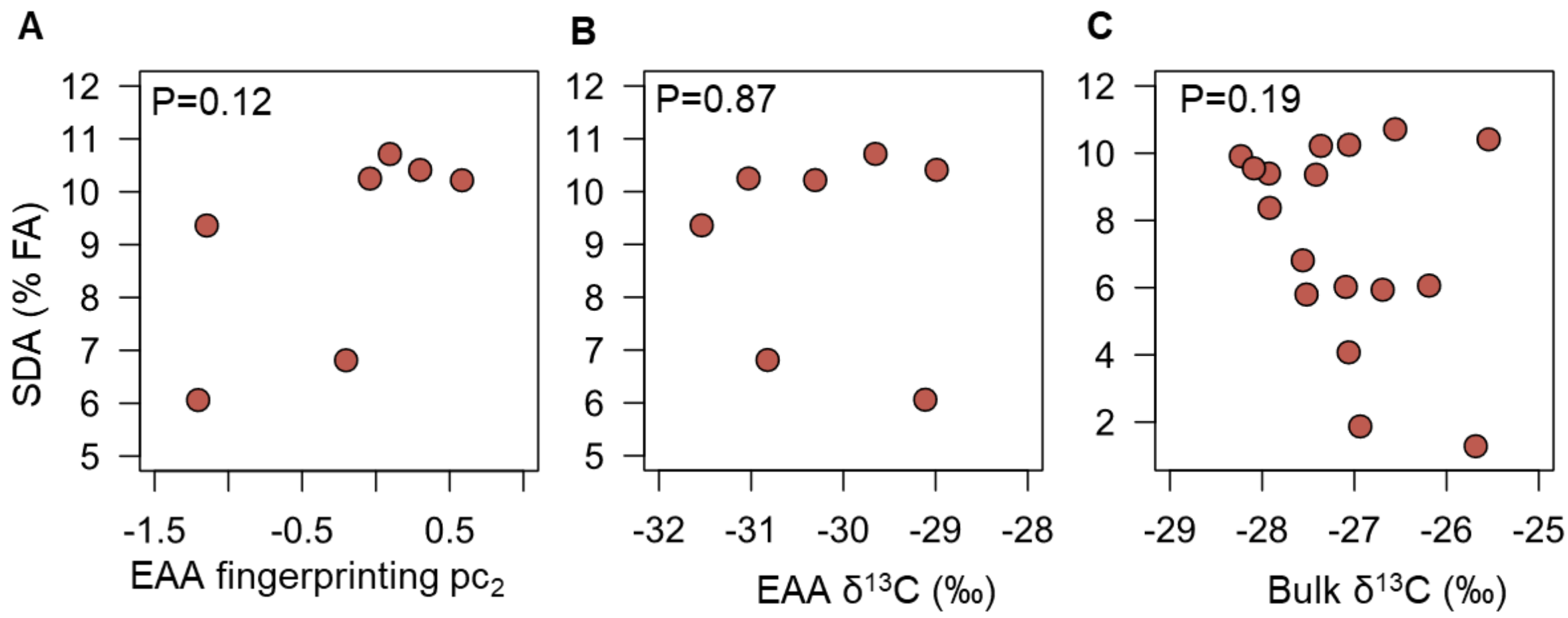

Figure S17. Relationship between SDA relative abundance and dietary markers. Relationship between SDA relative abundance (% FA) in individual char from the glacial lake without fjord connection, Qeqertaasaq Tasia, and (A) essential amino acid (EAA) $\delta^{13}C$ fingerprinting, as inferred from principal component analysis axis 2, which showed the strongest differentiation (Fig. 3A), (B) EAA $\delta^{13}C$, and (C) bulk $\delta^{13}C$. P-values are from linear regressions.

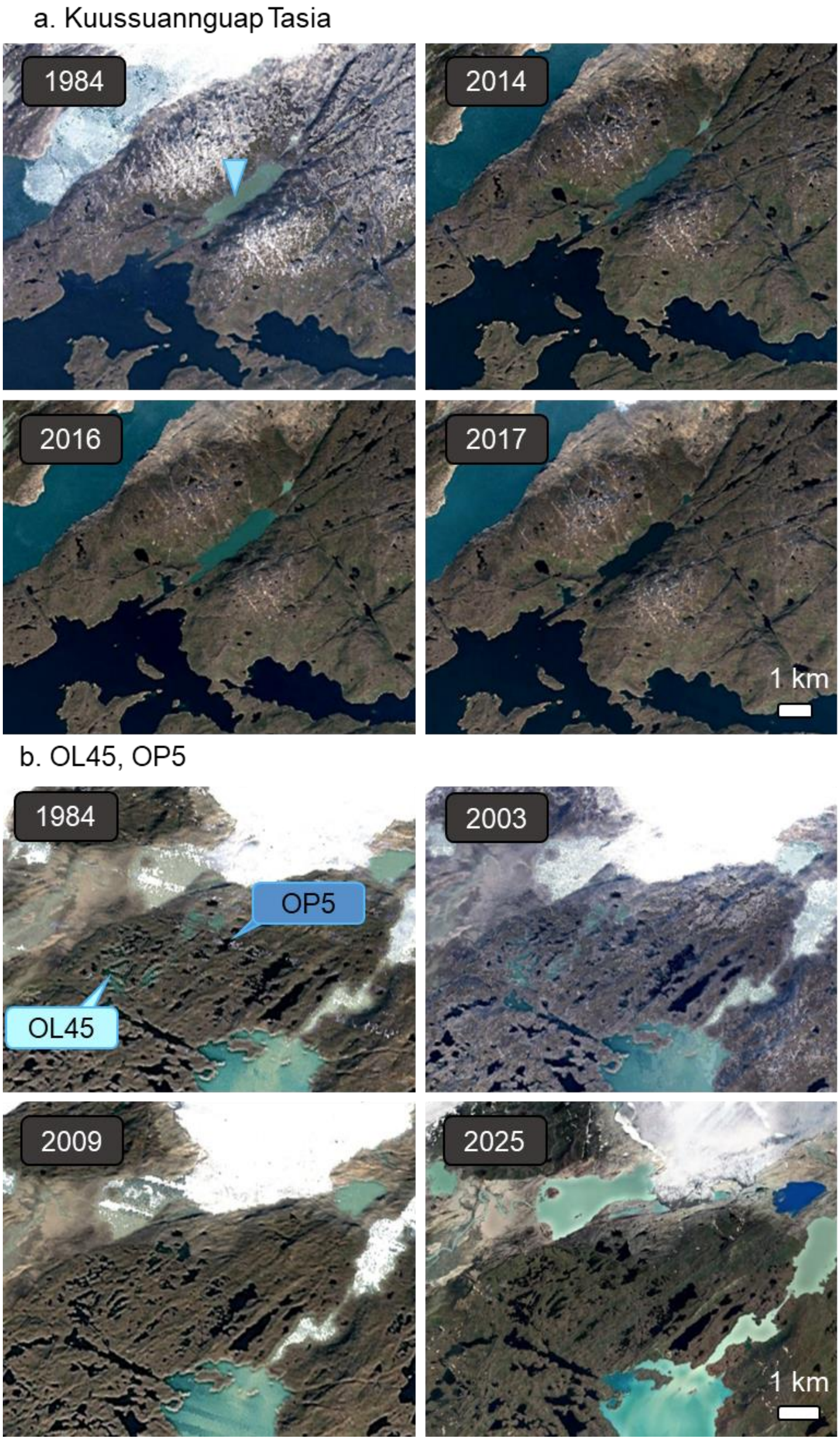

Figure S18. Satellite imaging of glacial disconnection of lakes. Satellite imagery from Google Earth (image: © 2026 Airbus; © U.S. Geological Survey; Landsat/Copernicus data; IBCAO), showing the three recent lakes, (a) Kuussuannguap Tasia, (b) OL45 and OP5, that have undergone recent deglaciation following modern warming. Arrows indicate lakes. Each panel presents a time series illustrating the transition from proglacial to non-glacial lacustrine conditions, visible as a marked shift in lake color from turbid brown to clear dark blue. The transitions occurred after 2016 for Kuusuanngup Tasia and after 2003 for OL45. OP5 already appeared non-glacial in the earliest available imagery.

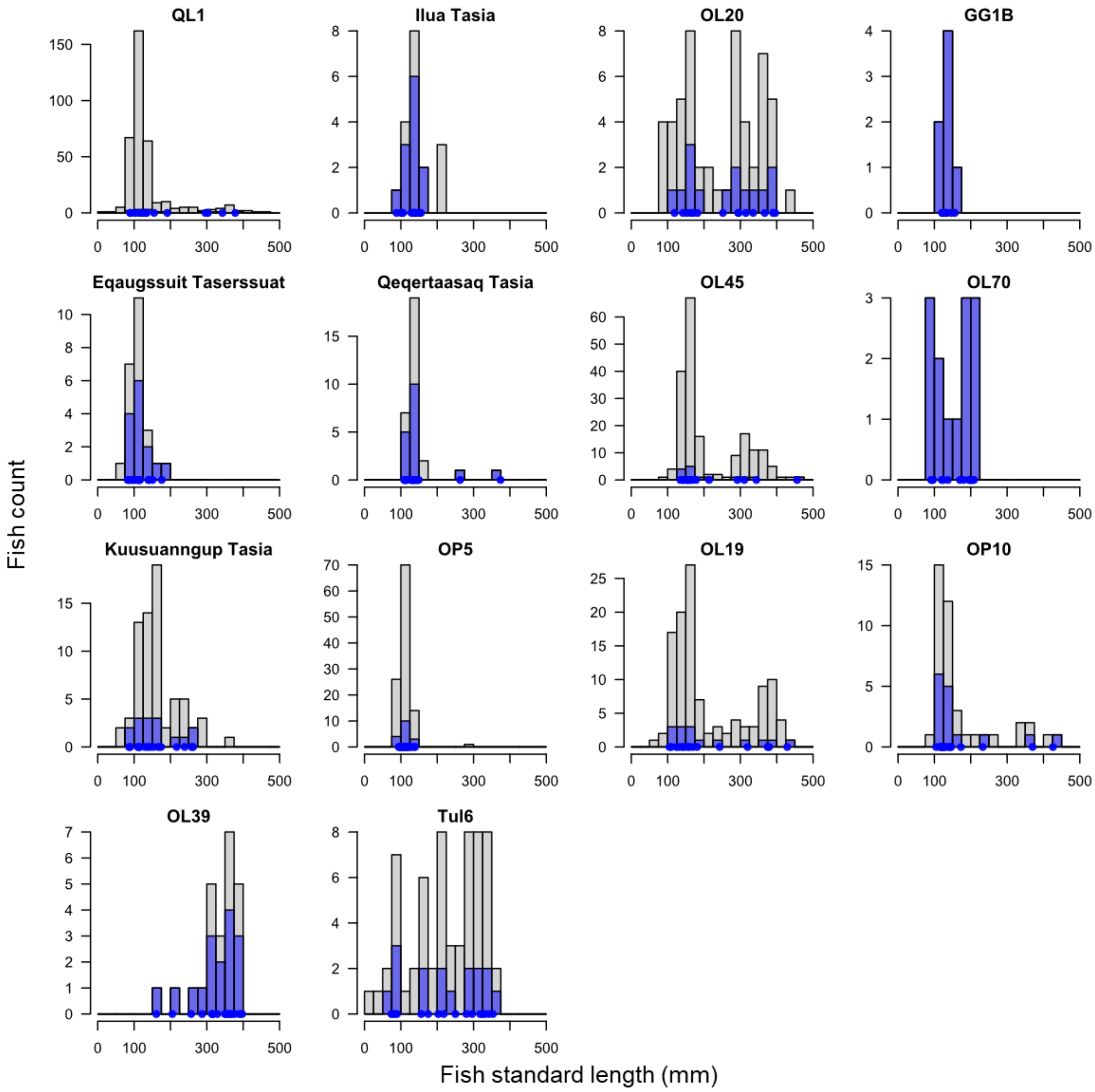

Figure S19. Selection of resident char for fatty acid analyses. Sample selection for bulk isotope and FA analyses. Histograms show the size distribution of resident char captured in the different lakes. The overlaid blue histograms indicate the distribution of individuals selected for muscle tissue analysis of stable isotopes and fatty acids. Blue points mark the selected individuals. Note the particularly large sample size for QL1, which reflects additional sampling conducted as part of other studies.

TABLES

Table S1. Summary of the PERMANOVA analysis testing differences among lake categories defined by the combination of lake stage (1 - Glacial, 2 - Recent, and 3 - Old) and fjord access (no migration: −mig; migration: +mig), as well as anadromous char treated as a separate group. The summary includes R^2, p-value (P_value), and adjusted p-value (P_adj), with Holm corrections applied.

	bulk			fatty acids		
**Comparison**	**R^2**	**P_value**	**P_adj**	**R^2**	**P_value**	**P_adj**
1 - Glacial - mig vs 1 - Glacial + mig	0.39	0.001	0.021	0.23	0.001	0.021
1 - Glacial - mig vs 2 - Recent - mig	0.76	0.001	0.021	0.42	0.001	0.021
1 - Glacial - mig vs 2 - Recent + mig	0.84	0.001	0.021	0.43	0.001	0.021
1 - Glacial - mig vs 3 - Old - mig	0.33	0.001	0.021	0.32	0.001	0.021
1 - Glacial - mig vs 3 - Old + mig	0.21	0.001	2.000	0.21	0.001	0.021
1 - Glacial - mig vs Anadromous	0.96	0.001	0.021	0.46	0.001	0.021
1 - Glacial + mig vs 2 - Recent - mig	0.24	0.001	0.021	0.38	0.001	0.021
1 - Glacial + mig vs 2 - Recent + mig	0.20	0.002	0.021	0.06	0.020	0.040
1 - Glacial + mig vs 3 - Old - mig	0.07	0.008	0.021	0.24	0.001	0.021
1 - Glacial + mig vs 3 - Old + mig	0.09	0.002	0.021	0.14	0.001	0.021
1 - Glacial + mig vs Anadromous	0.35	0.001	0.021	0.32	0.001	0.021
2 - Recent - mig vs 2 - Recent + mig	0.73	0.001	0.021	0.43	0.001	0.021
2 - Recent - mig vs 3 - Old - mig	0.31	0.001	0.021	0.10	0.001	0.021
2 - Recent - mig vs 3 - Old + mig	0.34	0.001	0.021	0.09	0.001	0.021
2 - Recent - mig vs Anadromous	0.86	0.001	0.021	0.51	0.001	0.021
2 - Recent + mig vs 3 - Old - mig	0.44	0.001	0.021	0.20	0.001	0.021
2 - Recent + mig vs 3 - Old + mig	0.37	0.001	0.021	0.12	0.001	0.021
2 - Recent + mig vs Anadromous	0.29	0.001	0.021	0.40	0.001	0.021
3 - Old - mig vs 3 - Old + mig	0.02	0.205	0.205	0.02	0.070	0.070
3 - Old - mig vs Anadromous	0.65	0.001	0.021	0.39	0.001	0.021
3 - Old + mig vs Anadromous	0.58	0.001	0.021	0.32	0.001	0.021

Table S2. Summary of linear mixed-effects model selection for bulk $\delta^{15}N$, including lake as a random factor. The table presents the potential explanatory variables: catch per unit effort of char (CPUE), used as a proxy for total char production; lake size (Size); char standard length (SL); lake stage (Stage); and their one-way interactions (symbol ":"). Values indicate the effect of each variable, while "+" indicates that the categorical variable was retained in the model. "NA" indicates that the variable was not retained. The following statistics are reported: df (degrees of freedom), LogLik (log-likelihood), AICc (corrected Akaike Information Criterion), ΔAICc (difference from the best model, i.e., the model with the lowest AICc), and weight (model weight). The table reports all models with ΔAICc < 30; the full table can be found in the data repository.

Intercept	CPUE	Size	SL	Stage	CPUE : Size	CPUE : SL	CPUE : Stage	Size: SL	Size : Stage	SL : Stage	df	logLik	AICc	delta	weight
6.18	NA	NA	0.01	+	NA	NA	NA	NA	NA	NA	6.00	-185.19	383.01	0.00	0.93
6.41	NA	NA	0.01	NA	NA	NA	NA	NA	NA	NA	4.00	-190.11	388.52	5.51	0.06
6.19	0.00	NA	0.01	+	NA	NA	NA	NA	NA	NA	7.00	-189.47	393.79	10.78	0.00
6.04	NA	0.00	0.01	+	NA	NA	NA	NA	NA	NA	7.00	-190.69	396.24	13.23	0.00
6.28	NA	0.00	0.01	NA	NA	NA	NA	NA	NA	NA	5.00	-195.09	400.63	17.61	0.00
6.55	0.00	NA	0.01	NA	NA	NA	NA	NA	NA	NA	5.00	-195.10	400.66	17.65	0.00
7.18	NA	NA	0.01	+	NA	NA	NA	NA	NA	+	8.00	-192.40	401.90	18.89	0.00
6.17	0.00	NA	0.01	+	NA	NA	+	NA	NA	NA	8.00	-193.21	403.53	20.52	0.00
6.04	NA	0.00	0.01	+	NA	NA	NA	NA	+	NA	8.00	-193.69	404.48	21.47	0.00
5.91	0.00	0.00	0.01	+	NA	NA	NA	NA	NA	NA	8.00	-194.41	405.93	22.92	0.00
6.33	0.00	0.00	0.01	NA	NA	NA	NA	NA	NA	NA	6.00	-199.98	412.60	29.59	0.00

Table S3. Summary of generalized linear model selection for the different response variables: LC-PUFAs, ARA, DHA, and EPA concentration (mg g^{-1} dry weight). The table presents the potential explanatory variables: fjord access (anadromy = yes), lake stage, char standard length (SL), and their interaction (symbol ":"). Values indicate the effect of each variable, while "+" indicates that the categorical variable was retained in the model. "NA" indicates that the variable was not retained. The following statistics are reported: df (degrees of freedom), LogLik (log-likelihood), AICc (corrected Akaike Information Criterion), ΔAICc (difference from the best model, i.e., the model with the lowest AICc), and weight (model weight). The table reports all models with ΔAICc < 30; the full table can be found in the data repository.

Response variable	(Intercept)	Anadromy	SL	Stage	Anadromy : SL	Anadromy : Stage	SL : Stage	df	logLik	AICc	delta	weight
**LC-PUFAs**	**11.20**	**+**	**-0.01**	**+**	**NA**	**+**	**NA**	**8.00**	**-587.09**	**1190.95**	**0.00**	**0.59**
LC-PUFAs	11.51	+	-0.02	+	+	+	NA	9.00	-586.92	1192.80	1.85	0.24
LC-PUFAs	12.38	+	-0.02	+	NA	+	+	10.00	-586.45	1194.08	3.13	0.12
LC-PUFAs	12.56	+	-0.02	+	+	+	+	11.00	-586.38	1196.19	5.24	0.04
LC-PUFAs	9.11	+	NA	+	NA	+	NA	7.00	-592.93	1200.46	9.51	0.01
**DHA**	**6.54**	**+**	**0.00**	**+**	**NA**	**+**	**NA**	**8.00**	**-501.84**	**1020.45**	**0.00**	**0.48**
DHA	5.79	+	NA	+	NA	+	NA	7.00	-503.67	1021.93	1.47	0.23
DHA	6.73	+	-0.01	+	+	+	NA	9.00	-501.69	1022.34	1.89	0.19
DHA	6.39	+	0.00	+	NA	+	+	10.00	-501.55	1024.29	3.84	0.07
DHA	6.52	+	0.00	+	+	+	+	11.00	-501.48	1026.38	5.93	0.02
DHA	8.19	+	0.00	+	+	NA	NA	7.00	-528.49	1071.57	51.11	0.00
**ARA**	**0.47**	**+**	**NA**	**+**	**NA**	**+**	**NA**	**7.00**	**-226.41**	**467.41**	**0.00**	**0.30**
ARA	0.70	+	0.00	+	+	+	NA	9.00	-224.40	467.76	0.35	0.25
ARA	1.15	+	0.00	+	+	+	+	11.00	-222.63	468.69	1.28	0.16
ARA	1.03	+	0.00	+	NA	+	+	10.00	-223.81	468.80	1.39	0.15
ARA	0.54	+	0.00	+	NA	+	NA	8.00	-226.14	469.04	1.64	0.13
ARA	1.82	+	-0.01	+	NA	NA	+	8.00	-234.19	485.14	17.74	0.00
**EPA**	**3.55**	**+**	**-0.01**	**+**	**NA**	**+**	**NA**	**8.00**	**-340.58**	**697.93**	**0.00**	**0.61**
EPA	3.50	+	-0.01	+	+	+	NA	9.00	-340.53	700.03	2.10	0.21
EPA	4.03	+	-0.01	+	NA	+	+	10.00	-340.22	701.63	3.70	0.10
EPA	3.98	+	-0.01	+	+	+	+	11.00	-340.15	703.73	5.80	0.03
EPA	4.39	NA	-0.01	NA	NA	NA	NA	3.00	-349.45	705.02	7.10	0.02
EPA	3.95	+	-0.01	NA	+	NA	NA	5.00	-347.59	705.50	7.57	0.01

Table S4. Summary of generalized linear model selection for DHA relative abundance (% FA). The table presents the explanatory variables: detrital contribution (det), as inferred from bacterial FAs (C15:0 + C17:0; % FA), relative change trophic position, as inferred from $\delta^{15}N$ corrected for marine reliance (dN_res), metabolic regulation, as depicted by SDA (% FA) (SDA), char standard length (SL), and the interaction between SL and SDA with Stage. Values indicate the effect of each variable, while "+" indicates that the categorical variable was retained in the model. "NA" indicates that the variable was not retained. The following statistics are reported: df (degrees of freedom), LogLik (log-likelihood), AICc (corrected Akaike Information Criterion), ΔAICc (difference from the best model, i.e., the model with the lowest AICc), and weight (model weight). The table reports all models with ΔAICc < 30; the full table can be found in the data repository.

Intercept	det	dN_res	Mar	SDA	SL	SDA:Stage	SL:Stage	df	logLik	AICc	delta	weight
37.8	-13.0	1.7	12.3	-1.2	0.0	+	NA	9.0	-597.1	1213.2	0.0	0.8
37.4	-12.4	1.8	11.8	-1.3	0.0	+	+	11.0	-596.2	1215.9	2.6	0.2
36.6	-13.3	2.0	11.5	-1.5	0.0	NA	+	9.0	-601.4	1221.7	8.5	0.0
37.2	-13.7	1.5	13.0	-1.3	0.0	NA	NA	7.0	-606.7	1228.0	14.8	0.0
35.6	-13.3	0.6	10.4	-1.3	NA	+	NA	8.0	-606.2	1229.2	15.9	0.0
36.7	-12.8	NA	8.9	-1.4	NA	+	NA	7.0	-608.4	1231.3	18.1	0.0
37.8	-12.5	NA	8.6	-1.4	0.0	+	NA	8.0	-607.4	1231.6	18.3	0.0
38.2	-12.9	NA	8.8	-1.4	0.0	+	+	10.0	-607.2	1235.5	22.3	0.0
35.2	-14.1	0.4	11.2	-1.3	NA	NA	NA	6.0	-613.8	1240.0	26.8	0.0
36.5	-14.6	NA	9.6	-1.5	NA	NA	NA	5.0	-615.3	1240.8	27.6	0.0
37.7	-14.7	NA	9.2	-1.5	0.0	NA	NA	6.0	-614.5	1241.4	28.2	0.0

Table S5. Summary of the 14 study lakes, including successional stage classification, total sampled resident char, total analyzed (FA and bulk isotopes) resident char, geographic coordinates, hydrological connection to the fjord (Yes: anadromy is possible, No: migration barrier prevents upstream migration), morphometric characteristics, water clarity (Secchi depth), deglaciation age, satellite-based assessment of recent deglaciation using Google Earth, and estimated timing of isolation from the sea. References correspond to sources used for geological history: a (*96*), b (*97*), c (*98*), d (*72*), e (*30*) and f (*99*).

Lake name	Stage	Total resident char sampled	Total resident char analyzed	GPS_N	GPS_W	Access to the fjord	Maximum depth (m)	Size (HA)	Secchi depth (m)	Deglaciation (ka)	Ref	Recent deglaciation (Google Earth)	Isolation from sea (ka)	Ref
Ilua Tasia	3- Old	18	12	60.97	-45.78	Yes	12.3	66.9	NA	After 11.4	a		~ 9.1	b
OL19	3- Old	115	15	60.99	-47.51	No	27.8	42.2	6.5	11 to 10.5 (min lim. 9.5-7.9)	d		After 10.0	e
OL20	3- Old	54	14	60.99	-47.52	Yes	16.9	60.8	6.5	11 to 10.5 (min lim. 9.5-7.9)	d		After 10.0	e
OL39	3- Old	24	16	60.96	-47.45	No	8	25	NA	11.5 to 11 (min lim. 9.5-7.9)	d		After 10.0	e
OP10	3- Old	41	15	60.97	-47.45	No	12.4	13.3	NA	11.5 to 11 (min lim. 9.5-7.9)	d		After 10.0	e
Qaqortoq Tasermiut	3- Old	355	14	60.73	-46.07	Yes	50	322	NA	12.3 to 11.4	a		After 11.0-9.0	f
Tul6	3- Old	62	16	60.85	-46.45	Yes	26	29	NA	12.3 to 11.4	a		~ 9.1	b
Kuussuannguap Tasia	2- Recent	69	15	60.96	-46.91	Yes	94.5	161	10	11.5 to 11 (min lim. 8.4-7.9)	d	After 2016	Above local marine limit	
OL45	2- Recent	190	15	60.99	-47.59	No	11	38.3	4.5	11.5 to 11 (min lim. 9.5-7.9)	d	After 2003	After 10.0	e
OP5	2- Recent	111	17	61.00	-47.54	No	5.7	10.7	4.5	11 to 10.5 (min lim. 9.5-7.9)	d	Before 1985	After 9.0	e
Eqalussuit Tasersuat	1- Proglacial	29	14	61.02	-47.41	Yes	66	628	0.1	11 to 10.5 (min lim. 9.5-7.9)	d		Above local marine limit	
GG1B	1- Proglacial	7	7	61.02	-47.57	Yes	34.2	255	0.1	11 to 10.5 (min lim. 9.5 -7.9)	d		After 10.0	e
OL70	1- Proglacial	13	13	60.99	-47.48	Yes	14.5	93.2	0.2	11 to 10.5 (min lim. 9.5 -7.9)	d		After 10.0	e
Qeqertaasaq Tasia	1- Proglacial	30	17	61.11	-46.13	No	23.4	166	0.2	11.3 to 8.8	c		~ 7.6	f

Captions for the supplementary table files

Table S1. Summary of the PERMANOVA analysis testing differences among lake categories defined by the combination of lake stage (1 - Glacial, 2 - Recent, and 3 - Old) and fjord access (no migration: −mig; migration: +mig), as well as anadromous char treated as a separate group. The summary includes R^2, p-value (P_value), and adjusted p-value (P_adj), with Holm corrections applied.

Table S2. Summary of linear mixed-effects model selection for bulk $\delta^{15}N$, including lake as a random factor. The table presents the potential explanatory variables: catch per unit effort of char (CPUE), used as a proxy for total char production; lake size (Size); char standard length (SL); lake stage (Stage); and their one-way interactions (symbol ":"). Values indicate the effect of each variable, while "+" indicates that the categorical variable was retained in the model. "NA" indicates that the variable was not retained. The following statistics are reported: df (degrees of freedom), LogLik (log-likelihood), AICc (corrected Akaike Information Criterion), ΔAICc (difference from the best model, i.e., the model with the lowest AICc), and weight (model weight). The table reports all models with ΔAICc < 30; the full table can be found in the data repository.

Table S3. Summary of generalized linear model selection for the different response variables: LC-PUFAs, ARA, DHA, and EPA concentration (mg g^{-1} dry weight). The table presents the potential explanatory variables: fjord access (anadromy = yes), lake stage, char standard length (SL), and their interaction (symbol ":"). Values indicate the effect of each variable, while "+" indicates that the categorical variable was retained in the model. "NA" indicates that the variable was not retained. The following statistics are reported: df (degrees of freedom), LogLik (log-likelihood), AICc (corrected Akaike Information Criterion), ΔAICc (difference from the best model, i.e., the model with the lowest AICc), and weight (model weight). The table reports all models with ΔAICc < 30; the full table can be found in the data repository.

Table S4. Summary of generalized linear model selection for DHA relative abundance (% FA). The table presents the explanatory variables: detrital contribution (det), as inferred from bacterial FAs (C15:0 + C17:0; % FA), relative change trophic position, as inferred from $\delta^{15}N$ corrected for marine reliance (dN_res), metabolic regulation, as depicted by SDA (% FA) (SDA), char standard length (SL), and the interaction between SL and SDA with Stage. Values indicate the effect of each variable, while "+" indicates that the categorical variable was retained in the model. "NA" indicates that the variable was not retained. The following statistics are reported: df (degrees of freedom), LogLik (log-likelihood), AICc (corrected Akaike Information Criterion), ΔAICc (difference from the best model, i.e., the model with the lowest AICc), and weight (model weight). The table reports all models with ΔAICc < 30; the full table can be found in the data repository.

Table S5. Summary of the 14 study lakes, including successional stage classification, total sampled resident char, total analyzed (FA and bulk isotopes) resident char, geographic coordinates, hydrological connection to the fjord (Yes: anadromy is possible, No: migration barrier prevents upstream migration), morphometric characteristics, water clarity (Secchi depth), deglaciation age, satellite-based assessment of recent deglaciation using Google Earth, and estimated timing of isolation from the sea. References correspond to sources used for geological history: a (*96*), b (*97*), c (*98*), d (*72*), e (*30*) and f (*99*).

REFERENCES

1. A. Bliss, R. Hock, V. Radić, Global response of glacier runoff to twenty-first century climate change. *J. Geophys. Res. Earth Surf.* **119**, 717–730 (2014).

2. J.-B. Bosson, M. Huss, S. Cauvy-Fraunié, J.-C. Clément, G. Costes, M. Fischer, J. Poulenard, F. Arthaud, Future emergence of new ecosystems caused by glacial retreat. *Nature* **620**, 562–569 (2023).

3. D. H. Shugar, A. Burr, U. K. Haritashya, J. S. Kargel, C. S. Watson, M. C. Kennedy, A. R. Bevington, R. A. Betts, S. Harrison, K. Strattman, Rapid worldwide growth of glacial lakes since 1990. *Nat. Clim. Change* **10**, 939–945 (2020).

4. J. L. Carrivick, P. How, J. M. Lea, J. L. Sutherland, M. Grimes, F. S. Tweed, S. Cornford, D. J. Quincey, J. Mallalieu, Ice-marginal proglacial lakes across Greenland: Present status and a possible future. *Geophys. Res. Lett.* **49**, e2022GL099276 (2022).

5. K. C. Rose, D. P. Hamilton, C. E. Williamson, C. G. McBride, J. M. Fischer, M. H. Olson, J. E. Saros, M. G. Allan, N. Cabrol, Light attenuation characteristics of glacially-fed lakes. *J. Geophys. Res. Biogeosci.* **119**, 1446–1457 (2014).

6. P. R. Leavitt, B. F. Cumming, J. P. Smol, M. Reasoner, R. Pienitz, D. A. Hodgson, Climatic control of ultraviolet radiation effects on lakes. *Limnol. Oceanogr.* **48**, 2062–2069 (2003).

7. R. Sommaruga, G. Kandolf, Negative consequences of glacial turbidity for the survival of freshwater planktonic heterotrophic flagellates. *Sci. Rep.* **4**, 4113 (2014).

8. E. Jeppesen, T. A. Davidson, M. Meerhoff, L. De Meester, I. González-Bergonzoni, N. Vidal, H. Arndt, K. Jürgens, R. Sommaruga, K. Özkan, T. L. Lauridsen, S. Tserenpil, Differences in food web structure and composition between new and nearby older lakes in West Greenland suggest succession trajectories driven by glacier retreat. *Hydrobiologia* **850**, 4745–4761 (2023).

9. S. Cauvy-Fraunié, O. Dangles, A global synthesis of biodiversity responses to glacier retreat. *Nat. Ecol. Evol.* **3**, 1675–1685 (2019).

10. K. J. Pitman, J. W. Moore, M. Huss, M. R. Sloat, D. C. Whited, T. J. Beechie, R. Brenner, E. W. Hood, A. M. Milner, G. R. Pess, Glacier retreat creating new Pacific salmon habitat in western North America. *Nat. Commun.* **12**, 6816 (2021).

11. R. Tiberti, F. Buscaglia, C. Callieri, M. Rogora, G. Tartari, R. Sommaruga, Food web complexity of high mountain lakes is largely affected by glacial retreat. *Ecosystems* **23**, 1093–1106 (2020).

12. A. López-Rodríguez, E. Jeppesen, T. A. Davidson, L. D. Meester, I. González-Bergonzoni, N. Vidal, R. Sommaruga, K. Özkan, H. Arndt, K. Jürgens, M. Meerhoff, Ecosystem age drives food web architecture of glacier retreat-formed fishless ponds in Greenland. *Oikos*, **2026**, e11415 (2026).

13. C. J. Doenz, A. K. Krähenbühl, J. Walker, O. Seehausen, J. Brodersen, Ecological opportunity shapes a large Arctic charr species radiation. *Proc. R. Soc. B Biol. Sci.* **286**, 20191992 (2019).

14. K. Prach, L. R. Walker, Four opportunities for studies of ecological succession. *Trends Ecol. Evol.* **26**, 119–123 (2011).

15. F. S. Chapin, L. R. Walker, C. L. Fastie, L. C. Sharman, Mechanisms of primary succession following deglaciation at Glacier Bay, Alaska. *Ecol. Monogr.* **64**, 149–175 (1994).

16. N. J. Mantua, H. Bell, A. E. Todgham, M. E. Daniels, J. Rinchard, J. M. Ludwig, J. C. Field, S. T. Lindley, F. E. Rowland, C. A. Richter, D. Walters, B. Finney, H. A. R. Distajo, D. Tillitt, D. C. Honeyfield, T. Lipscomb, K. Kwak, J. Kindopp, D. Cocherell, A. Ward, T. H. Williams, J. Harding, N. A. Fangue, C. Jeffres, R. I. Ruiz-Cooley, S. Y. Litvin, S. Foott, M. Adkison, B. Kormos, P. Harte, F. Colwell, C. P. Suffridge, K. C. Shannon, A. Cranford, C. Ambrose, A. Reed, R. C. Johnson, Widespread thiamine deficiency in California salmon linked to an anchovy-dominated marine prey base. *Proc. Natl. Acad. Sci. U.S.A.* **122**, e2426011122 (2025).

17. S. D. Newsome, M. L. Fogel, L. Kelly, C. M. del Rio, Contributions of direct incorporation from diet and microbial amino acids to protein synthesis in Nile tilapia: Contributions of diet and microbial amino acids to protein synthesis. *Funct. Ecol.* **25**, 1051–1062 (2011).

18. D. C. Müller-Navarra, M. T. Brett, A. M. Liston, C. R. Goldman, A highly unsaturated fatty acid predicts carbon transfer between primary producers and consumers. *Nature* **403**, 74–77 (2000).

19. C. W. Twining, J. T. Brenna, N. G. Hairston, A. S. Flecker, Highly unsaturated fatty acids in nature: What we know and what we need to learn. *Oikos* **125**, 749–760 (2016).

20. C. W. Twining, J. T. Brenna, P. Lawrence, J. R. Shipley, T. N. Tollefson, D. W. Winkler, Omega-3 long-chain polyunsaturated fatty acids support aerial insectivore performance more than food quantity. *Proc. Natl. Acad. Sci. U.S.A.* **113**, 10920–10925 (2016).

21. S. J. Taipale, K. Pulkkinen, O. Keva, M. J. Kainz, H. Nykänen, Lowered nutritional quality of prey decrease the growth and biomolecule content of rainbow trout fry. *Comp. Biochem. Physiol. B Biochem. Mol. Biol.* **262**, 110767 (2022).

22. S. Mari, S. Auer, B. Austad, P. Hansson, S. Vitecek, M. J. Yegon, L. Závorka, Dietary omega-3 long-chain polyunsaturated fatty acids can enhance ecologically relevant cognitive traits in juvenile brown trout. *Ecol. Evol.* **15**, e72340 (2025).

23. N. Kabeya, M. M. Fonseca, D. E. Ferrier, J. C. Navarro, L. K. Bay, D. S. Francis, D. R. Tocher, L. F. C. Castro, Ó. Monroig, Genes for de novo biosynthesis of omega-3 polyunsaturated fatty acids are widespread in animals. *Sci. Adv.* **4**, eaar6849 (2018).

24. D. R. Engstrom, S. C. Fritz, J. E. Almendinger, S. Juggins, Chemical and biological trends during lake evolution in recently deglaciated terrain. *Nature* **408**, 161–166 (2000).

25. S. C. Fritz, N. J. Anderson, The relative influences of climate and catchment processes on Holocene lake development in glaciated regions. *J. Paleolimnol.* **49**, 349–362 (2013).

26. L. Závorka, A. Blanco, F. Chaguaceda, J. Cucherousset, S. S. Killen, C. Liénart, M. Mathieu-Resuge, P. Němec, M. Pilecky, K. Scharnweber, The role of vital dietary biomolecules in eco-evo-devo dynamics. *Trends Ecol. Evol.* **38**, 72–84 (2023).

27. A. Ishikawa, N. Kabeya, K. Ikeya, R. Kakioka, J. N. Cech, N. Osada, M. C. Leal, J. Inoue, M. Kume, A. Toyoda, A. Tezuka, A. J. Nagano, Y. Y. Yamasaki, Y. Suzuki, T. Kokita, H. Takahashi, K. Lucek, D. Marques, Y. Takehana, K. Naruse, S. Mori, O. Monroig, N. Ladd, C. J. Schubert, B. Matthews, C. L. Peichel, O. Seehausen, G. Yoshizaki, J. Kitano, A key metabolic gene for recurrent freshwater colonization and radiation in fishes. *Science* **364**, 886–889 (2019).

28. O. Keva, S. J. Taipale, B. Hayden, S. M. Thomas, J. Vesterinen, P. Kankaala, K. K. Kahilainen, Increasing temperature and productivity change biomass, trophic pyramids and community-level omega-3 fatty acid content in subarctic lake food webs. *Glob. Change Biol.* **27**, 282–296 (2021).

29. M. Sinensky, Homeoviscous adaptation—A homeostatic process that regulates the viscosity of membrane lipids in *Escherichia coli*. *Proc. Natl. Acad. Sci. U.S.A.* **71**, 522–525 (1974).

30. G. Luetzenburg, N. Ngoepe, W. R. Farnsworth, A. Rosenberg, E. Lewis, J. Brodersen, O. Bennike, M.-L. Siggaard-Andersen, S. Woodroffe, G. A. Milne, Relative sea-level changes and evidence for a Holocene low stand in southern Greenland. *Quat. Sci. Rev.* **374**, 109787 (2026).

31. T. Larsen, M. Ventura, N. Andersen, D. M. O'Brien, U. Piatkowski, M. D. McCarthy, Tracing carbon sources through aquatic and terrestrial food webs using amino acid stable isotope fingerprinting. *PLOS ONE* **8**, e73441 (2013).

32. E. Hood, T. J. Battin, J. Fellman, S. O'Neel, R. G. M. Spencer, Storage and release of organic carbon from glaciers and ice sheets. *Nat. Geosci.* **8**, 91–96 (2015).

33. G. M. Hilton, D. R. Thompson, P. M. Sagar, R. J. Cuthbert, Y. Cherel, S. J. Bury, A stable isotopic investigation into the causes of decline in a sub-Antarctic predator, the rockhopper penguin *Eudyptes chrysocome*. *Glob. Change Biol.* **12**, 611–625 (2006).

34. C. Laspoumaderes, B. Modenutti, M. S. Souza, M. Bastidas Navarro, F. Cuassolo, E. Balseiro, Glacier melting and stoichiometric implications for lake community structure: Zooplankton species distributions across a natural light gradient. *Glob. Change Biol.* **19**, 316–326 (2013).

35. G. Saboret, C. Moccetti, L. I. Wassenaar, B. Matthews, N. Jr. Aquino, D. J. Janssen, J. Brodersen, C. J. Schubert, Impact of glaciers on trophic dynamics and polyunsaturated fat accumulation in Southern Greenland Fjord ecosystems. *Glob. Change Biol.* **31**, e70044 (2025).

36. M. E. Sierszen, M. E. McDonald, D. A. Jensen, Benthos as the basis for arctic lake food webs. *Aquat. Ecol.* **37**, 437–445 (2003).

37. G. Grosbois, M. Power, M. Evans, G. Koehler, M. Rautio, Content, composition, and transfer of polyunsaturated fatty acids in an Arctic lake food web. *Ecosphere* **13**, e03881 (2022).

38. J. E. Saros, K. C. Rose, D. W. Clow, V. C. Stephens, A. B. Nurse, H. A. Arnett, J. R. Stone, C. E. Williamson, A. P. Wolfe, Melting alpine glaciers enrich high-elevation lakes with reactive nitrogen. *Environ. Sci. Technol.* **44**, 4891–4896 (2010).

39. H. Doi, H. Hillebrand, Historical contingency and productivity effects on food-chain length. *Commun. Biol.* **2**, 40 (2019).

40. D. M. Post, M. L. Pace, N. G. Hairston, Ecosystem size determines food-chain length in lakes. *Nature* **405**, 1047–1049 (2000).

41. B. B. Perren, N. J. Anderson, M. S. Douglas, S. C. Fritz, The influence of temperature, moisture, and eolian activity on Holocene lake development in West Greenland. *J. Paleolimnol.* **48**, 223–239 (2012).

42. J. E. Saros, V. Hazuková, R. M. Northington, G. P. Huston, A. Lamb, S. Birkel, R. Pereira, G. Bourdin, B. Jiang, S. McGowan, Abrupt transformation of west Greenland lakes following compound climate extremes associated with atmospheric rivers. *Proc. Natl. Acad. Sci. U.S.A.* **122**, e2413855122 (2025).

43. F. Chaguaceda, P. Eklöv, K. Scharnweber, Regulation of fatty acid composition related to ontogenetic changes and niche differentiation of a common aquatic consumer. *Oecologia* **193**, 325–336 (2020).

44. D. R. Tocher, Fatty acid requirements in ontogeny of marine and freshwater fish. *Aquacult. Res.* **41**, 717–732 (2010).

45. F. Guihéneuf, M. Fouqueray, V. Mimouni, L. Ulmann, B. Jacquette, G. Tremblin, Effect of UV stress on the fatty acid and lipid class composition in two marine microalgae *Pavlova lutheri* (Pavlovophyceae) and *Odontella aurita* (Bacillariophyceae). *J. Appl. Phycol.* **22**, 629–638 (2010).

46. M. Ilić, C. Werner, P. Fink, Equal relevance of omega-3 and omega-6 polyunsaturated fatty acids for the fitness of *Daphnia* spp. *Limnol. Oceanogr.* **64**, 2512–2525 (2019).

47. S. J. Taipale, E. Peltomaa, M. Hiltunen, R. I. Jones, M. W. Hahn, C. Biasi, M. T. Brett, Inferring phytoplankton, terrestrial plant and bacteria bulk $\delta^{13}C$ values from compound specific analyses of lipids and fatty acids. *PLOS ONE* **10**, e0133974 (2015).

48. T. Farkas, K. Kitajka, E. Fodor, I. Csengeri, E. Lahdes, Y. K. Yeo, Z. Krasznai, J. E. Halver, Docosahexaenoic acid-containing phospholipid molecular species in brains of vertebrates. *Proc. Natl. Acad. Sci. U.S.A.* **97**, 6362–6366 (2000).

49. L. Závorka, M. L. Wallerius, M. J. Kainz, J. Höjesjö, Linking omega-3 polyunsaturated fatty acids in natural diet with brain size of wild consumers. *Oecologia* **199**, 797–807 (2022).

50. M. Kainz, K. Telmer, A. Mazumder, Bioaccumulation patterns of methyl mercury and essential fatty acids in lacustrine planktonic food webs and fish. *Sci. Total Environ.* **368**, 271–282 (2006).

51. G. Saboret, B. J. W. Drost, C. Kowarik, C. J. Schubert, M. M. Gossner, M. Ilić, Quantifying the utilisation of blue, green and brown resources by riparian predators: A combined use of amino acid isotopes and fatty acids. *Methods Ecol. Evol.* **15**, 1450–1462 (2024).

52. S. M. Hixson, B. Sharma, M. J. Kainz, A. Wacker, M. T. Arts, Production, distribution, and abundance of long-chain omega-3 polyunsaturated fatty acids: A fundamental dichotomy between freshwater and terrestrial ecosystems. *Environ. Rev.* **23**, 414–424 (2015).

53. C. M. Hudson, C. W. Twining, M. Moosmann, R. Greenway, P. G. D. Feulner, B. Matthews, Prickly postglacial pioneers: Freshwater plankton community composition influences fatty acid desaturase (FADS2) copy number in southern Greenland threespine sticklebacks. *Oikos* **2025**, e10927 (2025).

54. M. K. Gregory, R. A. Gibson, R. J. Cook-Johnson, L. G. Cleland, M. J. James, Elongase reactions as control points in long-chain polyunsaturated fatty acid synthesis. *PLOS ONE* **6**, e29662 (2011).

55. J. G. Smith, J. Tomoleoni, M. Staedler, S. Lyon, J. Fujii, M. T. Tinker, Behavioral responses across a mosaic of ecosystem states restructure a sea otter–urchin trophic cascade. *Proc. Natl. Acad. Sci. U.S.A.* **118**, e2012493118 (2021).

56. J. R. Shipley, R. Oester, M. Mathieu-Resuge, T. P. Parmar, C. Kowarik, M. Ilíć, M. J. Kainz, D. Martin-Creuzburg, M. K. Obrist, C. H. Graham, M. M. Gossner, B. Matthews, C. W. Twining, Consumer biodiversity increases organic nutrient availability across aquatic and terrestrial ecosystems. *Science* **386**, 335–340 (2024).

57. B. B. Chapman, K. Hulthén, J. Brodersen, P. A. Nilsson, C. Skov, L.-A. Hansson, C. Brönmark, Partial migration in fishes: Causes and consequences. *J. Fish Biol.* **81**, 456–478 (2012).

58. R. J. Naiman, R. E. Bilby, D. E. Schindler, J. M. Helfield, Pacific salmon, nutrients, and the dynamics of freshwater and riparian ecosystems. *Ecosystems* **5**, 399–417 (2002).

59. M. D. Hocking, J. D. Reynolds, Impacts of salmon on riparian plant diversity. *Science* **331**, 1609–1612 (2011).

60. G. Saboret, C. Moccetti, K. Takatsu, D. J. Janssen, B. Matthews, J. Brodersen, C. J. Schubert, Glacial meltwater increases the dependence on marine subsidies of fish in freshwater ecosystems. *Ecosystems* **27**, 779–796 (2024).

61. J. W. Moore, D. E. Schindler, Nutrient export from freshwater ecosystems by anadromous sockeye salmon (*Oncorhynchus nerka*). *Can. J. Fish. Aquat. Sci.* **61**, 1582–1589 (2004).

62. C. Moccetti, N. Sperlich, G. Saboret, H. Ten Brink, J. Brodersen, Migratory-derived resources induce elongated food chains through middle-up food web effects. *Mov. Ecol.* **12**, 56 (2024).

63. J. Terborgh, J. A. Estes, *Trophic Cascades: Predators, Prey, and the Changing Dynamics of Nature* (Island Press, 2013).

64. R. L. Naylor, R. W. Hardy, D. P. Bureau, A. Chiu, M. Elliott, A. P. Farrell, I. Forster, D. M. Gatlin, R. J. Goldburg, K. Hua, P. D. Nichols, Feeding aquaculture in an era of finite resources. *Proc. Natl. Acad. Sci. U.S.A.* **106**, 15103–15110 (2009).

65. S. M. Hixson, M. T. Arts, Climate warming is predicted to reduce omega-3, long-chain, polyunsaturated fatty acid production in phytoplankton. *Glob. Change Biol.* **22**, 2744–2755 (2016).

66. B. D. Glencross, E. Bachis, M. B. Betancor, P. Calder, N. Liland, R. Newton, B. Ruyter, Omega-3 futures in aquaculture: Exploring the supply and demands for long-chain omega-3 essential fatty acids by aquaculture species. *Rev. Fish. Sci. Aquac.* **33**, 167–216 (2025).

67. N. J. Anderson, J. E. Saros, J. E. Bullard, S. M. P. Cahoon, S. McGowan, E. A. Bagshaw, C. D. Barry, R. Bindler, B. T. Burpee, J. L. Carrivick, R. A. Fowler, A. D. Fox, S. C. Fritz, M. E. Giles, L. Hamerlik, T. Ingeman-Nielsen, A. C. Law, S. H. Mernild, R. M. Northington, C. L. Osburn, S. Pla-Rabès, E. Post, J. Telling, D. A. Stroud, E. J. Whiteford, M. L. Yallop, J. C. Yde, The Arctic in the twenty-first century: Changing biogeochemical linkages across a paraglacial landscape of Greenland. *Bioscience* **67**, 118–133 (2017).

68. Y. Matsushita, K. Miyoshi, N. Kabeya, S. Sanada, R. Yazawa, Y. Haga, S. Satoh, Y. Yamamoto, C. A. Strüssmann, J. A. Luckenbach, Flatfishes colonised freshwater environments by acquisition of various DHA biosynthetic pathways. *Commun. Biol.* **3**, 516 (2020).

69. K. Bainour, N. Zulkifli, K.-K. Sam, J. C. Navarro, L. F. C. Castro, C. J. Glasby, A. C. Shu-Chien, Ó. Monroig, Freshwater-adapted polychaetes exhibit a complete enzymatic machinery for synthesizing long-chain polyunsaturated fatty acids. *Open Biol.* **15**, 250159 (2025).

70. M. Pilecky, L. Závorka, M. T. Arts, M. J. Kainz, Omega-3 PUFA profoundly affect neural, physiological, and behavioural competences – Implications for systemic changes in trophic interactions. *Biol. Rev.* **96**, 2127–2145 (2021).

71. F. Keck, T. Peller, R. Alther, C. Barouillet, R. Blackman, E. Capo, T. Chonova, M. Couton, L. Fehlinger, D. Kirschner, The global human impact on biodiversity. *Nature*, **641**, 395–400 (2025).

72. T. P. Leger, C. D. Clark, C. Huynh, S. Jones, J. C. Ely, S. L. Bradley, C. Diemont, A. L. Hughes, A Greenland-wide empirical reconstruction of paleo ice-sheet retreat informed by ice extent markers: PaleoGrIS version 1.0. *Clim. Past* **20**, 701–755 (2024).

73. S. E. Hampton, A. W. E. Galloway, S. M. Powers, T. Ozersky, K. H. Woo, R. D. Batt, S. G. Labou, C. M. O'Reilly, S. Sharma, N. R. Lottig, E. H. Stanley, R. L. North, J. D. Stockwell, R. Adrian, G. A. Weyhenmeyer, L. Arvola, H. M. Baulch, I. Bertani, L. L. Bowman, C. C. Carey, J. Catalan, W. Colom-Montero, L. M. Domine, M. Felip, I. Granados, C. Gries, H. Grossart, J. Haberman, M. Haldna, B. Hayden, S. N. Higgins, J. C. Jolley, K. K. Kahilainen, E. Kaup, M. J. Kehoe, S. MacIntyre, A. W. Mackay, H. L. Mariash, R. M. McKay, B. Nixdorf, P. Nõges, T. Nõges, M. Palmer, D. C. Pierson, D. M. Post, M. J. Pruett, M. Rautio, J. S. Read, S. L. Roberts, J. Rücker, S. Sadro, E. A. Silow, D. E. Smith, R. W. Sterner, G. E. A. Swann, M. A. Timofeyev, M. Toro, M. R. Twiss, R. J. Vogt, S. B. Watson, E. J. Whiteford, M. A. Xenopoulos, Ecology under lake ice. *Ecol. Lett.* **20**, 98–111 (2017).

74. M. E. Perga, D. Gerdeaux, 'Are fish what they eat' all year round? *Oecologia* **144**, 598–606 (2005).

75. J. Dalsgaard, M. St. John, G. Kattner, D. Müller-Navarra, W. Hagen, "Fatty acid trophic markers in the pelagic marine environment," in *Advances in Marine Biology* (Elsevier, 2003), vol. 46, pp. 225–340.

76. G. Saboret, D. Stalder, B. Matthews, J. Brodersen, C. J. Schubert, Autochthonous production sustains food webs in large perialpine lakes, independent of trophic status: Evidence from amino acid stable isotopes. *Freshw. Biol.* **68**, 870–887 (2023).

77. R Core Team, R: A language and environment for statistical computing, R Foundation for Statistical Computing (2020); www.R-project.org/).

78. vegan: Community ecology package, CRAN (2026); https://doi.org/10.32614/CRAN.package.vegan.

79. J. Fox, S. Weisberg, D. Adler, D. Bates, G. Baud-Bovy, S. Ellison, D. Firth, M. Friendly, G. Gorjanc, S. Graves, Package 'car'. *Vienna R Found. Stat. Comput.* **16**, 333 (2012).

80. F. De Mendiburu, R. Simon, Agricolae - Ten years of an open source statistical tool for experiments in breeding, agriculture and biology PeerJ 1404v1 [Preprint] (2015). https://doi.org/10.7287/peerj.preprints.1404v1.

81. B. Stock, B. Semmens, E. Ward, A. Parnell, A. Jackson, D. Phillips, Package 'MixSIAR': Bayesian mixing model R, version 3, CRAN (2018).

82. P. J. Manlick, S. D. Newsome, Stable isotope fingerprinting traces essential amino acid assimilation and multichannel feeding in a vertebrate consumer. *Methods Ecol. Evol.* **13**, 1819–1830 (2022).

83. J. H. Scott, D. M. O'Brien, D. Emerson, H. Sun, G. D. McDonald, A. Salgado, M. L. Fogel, An examination of the carbon isotope effects associated with amino acid biosynthesis. *Astrobiology* **6**, 867–880 (2006).

84. S. J. Iverson, C. Field, W. Don Bowen, W. Blanchard, Quantitative fatty acid signature analysis: A new method of estimating predator diets. *Ecol. Monogr.* **74**, 211–235 (2004).

85. J. Dawson, D. Pillay, R. Perissinotto, N. B. Richoux, Fatty acid analyses provide novel insights on hippo defecation and consequences for aquatic food webs. *Sci. Rep.* **10**, 12039 (2020).

86. Y. Chikaraishi, S. A. Steffan, N. O. Ogawa, N. F. Ishikawa, Y. Sasaki, M. Tsuchiya, N. Ohkouchi, High-resolution food webs based on nitrogen isotopic composition of amino acids. *Ecol. Evol.* **4**, 2423–2449 (2014).

87. C. J. Bradley, N. J. Wallsgrove, C. A. Choy, J. C. Drazen, E. D. Hetherington, D. K. Hoen, B. N. Popp, Trophic position estimates of marine teleosts using amino acid compound specific isotopic analysis: Stable isotope-derived trophic positions of teleosts. *Limnol. Oceanogr. Methods* **13**, 476–493 (2015).

88. D. M. Bates, *Lme4: Mixed-Effects Modeling with R* (Springer, 2010).

89. K. Barton, MuMIn: Multi-model inference. Httpr-Forge R-Proj. Orgprojectsmumin (2009).

90. S. R. Searle, F. M. Speed, G. A. Milliken, Population marginal means in the linear model: An alternative to least squares means. *Am. Stat.* **34**, 216–221 (1980).

91. J. Fox, S. Weisberg, M. Friendly, J. Hong, R. Andersen, D. Firth, S. Taylor, M. J. Fox, Package 'effects.' Url Httpwww R-Proj. Org Httpsocserv Socsci Mcmaster Cajfox (2016).

92. S. Jackman, A. Tahk, A. Zeileis, C. Maimone, J. Fearon, Z. Meers, M. S. Jackman, M. Imports, Package 'pscl'. *Polit. Sci. Comput. Lab.* **18** (2015).

93. M. V. Bell, D. R. Tocher, "Biosynthesis of polyunsaturated fatty acids in aquatic ecosystems: General pathways and new directions" in *Lipids in Aquatic Ecosystems*, M. Kainz, M. T. Brett, M. T. Arts, Eds. (Springer, 2009), pp. 211–236.

94. W. Revelle, M. W. Revelle, Package 'psych'. *Compr. R Arch. Netw.* **337**, 161–165 (2015).

95. C. L. Scott, S. Falk-Petersen, J. R. Sargent, H. Hop, O. J. Lønne, M. Poltermann, Lipids and trophic interactions of ice fauna and pelagic zooplankton in the marginal ice zone of the Barents Sea. *Polar Biol.* **21**, 65–70 (1999).

96. J. L. Andersen, D. L. Egholm, J. Olsen, N. K. Larsen, M. F. Knudsen, Topographical evolution and glaciation history of South Greenland constrained by paired 26Al/10Be nuclides. *Earth Planet. Sci. Lett.* **542**, 116300 (2020).

97. M. R. Kaplan, A. P. Wolfe, G. H. Miller, Holocene environmental variability in southern Greenland inferred from lake sediments. *Quatern. Res.* **58**, 149–159 (2002).

98. N. K. Larsen, J. Find, A. Kristensen, A. A. Bjørk, K. K. Kjeldsen, B. V. Odgaard, J. Olsen, K. H. Kjær, Holocene ice marginal fluctuations of the Qassimiut lobe in South Greenland. *Sci. Rep.* **6**, 22362 (2016).

99. C. J. Sparrenbom, O. Bennike, S. Björck, K. Lambeck, Relative sea-level changes since 15 000 cal. yr BP in the Nanortalik area, southern Greenland. *J. Quat. Sci.* **21**, 29–48 (2006).

100. L. I. E. Couturier, L. N. Michel, T. Amaro, S. M. Budge, E. da Costa, M. De Troch, V. Di Dato, P. Fink, C. Giraldo, F. Le Grand, I. Loaiza, M. Mathieu-Resuge, P. D. Nichols, C. C. Parrish, F. Sardenne, M. Vagner, F. Pernet, P. Soudant, State of art and best practices for fatty acid analysis in aquatic sciences. *ICES J. Mar. Sci.* **77**, 2375–2395 (2020).

101. D. M. Post, Using stable isotopes to estimate trophic position: Models, methods and assumptions. *Ecology* **83**, 703–718 (2002).

102. K. W. McMahon, Lipid extraction alters amino acid composition and bulk, but not amino acid, carbon and nitrogen isotope values. *Rapid Commun. Mass Spectrom.* **38**, e9848 (2024).

103. L. T. Corr, R. Berstan, R. P. Evershed, Development of *N*-acetyl methyl ester derivatives for the determination of $\delta^{13}C$ values of amino acids using gas chromatography-combustion-isotope ratio mass spectrometry. *Anal. Chem.* **79**, 9082–9090 (2007).

104. J. H. McCutchan, W. M. Lewis, C. Kendall, C. C. McGrath, Variation in trophic shift for stable isotope ratios of carbon, nitrogen, and sulfur. *Oikos* **102**, 378–390 (2003).

105. B. X. Semmens, E. J. Ward, J. W. Moore, C. T. Darimont, Quantifying inter- and intra-population niche variability using hierarchical bayesian stable isotope mixing models. *PLOS ONE* **4**, e6187 (2009).

106. B. C. Stock, B. X. Semmens, Unifying error structures in commonly used biotracer mixing models. *Ecology* **97**, 2562–2569 (2016).

107. K. Vane, M. R. D. Cobain, T. Larsen, The power and pitfalls of amino acid carbon stable isotopes for tracing origin and use of basal resources in food webs. *Ecol. Monogr.* **95**, e1647 (2025).

108. M. M. Pollierer, T. Larsen, A. Potapov, A. Brückner, M. Heethoff, J. Dyckmans, S. Scheu, Compound-specific isotope analysis of amino acids as a new tool to uncover trophic chains in soil food webs. *Ecological monographs* **89**, e01384 (2019).

109. M. D. Ramirez, A. C. Besser, S. D. Newsome, K. W. McMahon, Meta-analysis of primary producer amino acid $\delta^{15}N$ values and their influence on trophic position estimation. *Methods Ecol. Evol.* **12**, 1750–1767 (2021).

110. A.-N. Spiess, M. A.-N. Spiess, L. Rcpp, Package 'propagate,' CRAN (2018); www.verdeterreprod.fr/wp-content/uploads/2020/05/propagate.pdf.
